# Online Learning and Memory of Neural Trajectory Replays for Prefrontal Persistent and Dynamic Representations in the Irregular Asynchronous State

**DOI:** 10.3389/fncir.2021.648538

**Published:** 2021-07-08

**Authors:** Matthieu X. B. Sarazin, Julie Victor, David Medernach, Jérémie Naudé, Bruno Delord

**Affiliations:** ^1^Institut des Systèmes Intelligents et de Robotique, CNRS, Inserm, Sorbonne Université, Paris, France; ^2^CEA Paris-Saclay, CNRS, NeuroSpin, Saclay, France; ^3^Neuroscience Paris Seine - Institut de biologie Paris Seine, CNRS, Inserm, Sorbonne Université, Paris, France

**Keywords:** prefrontal cortex, neural trajectory, attractor, persistent and dynamical coding, working memory, learning, replay, asynchronous irregular state

## Abstract

In the prefrontal cortex (PFC), higher-order cognitive functions and adaptive flexible behaviors rely on continuous dynamical sequences of spiking activity that constitute neural trajectories in the state space of activity. Neural trajectories subserve diverse representations, from explicit mappings in physical spaces to generalized mappings in the task space, and up to complex abstract transformations such as working memory, decision-making and behavioral planning. Computational models have separately assessed learning and replay of neural trajectories, often using unrealistic learning rules or decoupling simulations for learning from replay. Hence, the question remains open of how neural trajectories are learned, memorized and replayed online, with permanently acting biological plasticity rules. The asynchronous irregular regime characterizing cortical dynamics in awake conditions exerts a major source of disorder that may jeopardize plasticity and replay of locally ordered activity. Here, we show that a recurrent model of local PFC circuitry endowed with realistic synaptic spike timing-dependent plasticity and scaling processes can learn, memorize and replay large-size neural trajectories online under asynchronous irregular dynamics, at regular or fast (sped-up) timescale. Presented trajectories are quickly learned (within seconds) as synaptic engrams in the network, and the model is able to chunk overlapping trajectories presented separately. These trajectory engrams last long-term (dozen hours) and trajectory replays can be triggered over an hour. In turn, we show the conditions under which trajectory engrams and replays preserve asynchronous irregular dynamics in the network. Functionally, spiking activity during trajectory replays at regular timescale accounts for both dynamical coding with temporal tuning in individual neurons, persistent activity at the population level, and large levels of variability consistent with observed cognitive-related PFC dynamics. Together, these results offer a consistent theoretical framework accounting for how neural trajectories can be learned, memorized and replayed in PFC networks circuits to subserve flexible dynamic representations and adaptive behaviors.

## Introduction

As when a few introductory notes recall a melody, in the immense space of known melodies, cerebral networks are able to memorize and replay complex temporal patterns in a flexible way. Such temporal patterns rely on continuous dynamical sequences of spiking activity, i.e., neural trajectories, that occur in recurrent neural networks of the prefrontal cortex (PFC) (Bakhurin et al., 2017; Paton and Buonomano, 2018; Wang et al., 2018). These neural trajectories emerge with learning, relying on dynamical engrams, which distinguish them from classical static engrams underlying Hebbian neuronal assemblies. In turn, these engrams likely arise through activity-dependent synaptic plasticity (Goto et al., 2010; Bittner et al., 2017). Hence, a robust understanding of the interplay between prefrontal dynamics and biological plastic processes is necessary to understand the emergence of functional neural trajectories and engrams. In the PFC of behaving animals, neural trajectories are embedded in an asynchronous and irregular background state activity that is markedly disordered (Destexhe et al., 2003; London et al., 2010). However, how synaptic plasticity builds engrams that are not erased by spontaneous activity and yet are not strong enough to alter irregular PFC dynamics remains an open question.

Neural trajectories correspond to organized spatio-temporal representations that peregrinate within the neural space (Shenoy et al., 2013). They are prominent in prefrontal cortices (Mante et al., 2013), where they subserve higher-order cognitive functions at diverse levels of abstraction (Wutz et al., 2018). In prefrontal areas, at the lowest levels of abstraction, neural trajectories can map the actual animal's position during effective trajectories within explicit spaces during visual perception (Mante et al., 2013) or navigation (Fujisawa et al., 2008; Zielinski et al., 2019). Beyond spatial mapping, neural trajectories can also depict generalized topological locations that are isomorphic to the task space, by multiplexing position, representation of goal locations and choice-related information (Fujisawa et al., 2008; Mashhoori et al., 2018; Yu et al., 2018; Kaefer et al., 2020). Neural trajectories have also been shown to subserve dynamical coding and manipulation of information during delay activities in working memory tasks involving the PFC (Lundqvist et al., 2018). In this context, neural trajectories do not represent explicit trajectories in external spaces, but implicit representations—of ongoing information and cognitive operations—that may prove useful for the task.

Rather than static maintenance of persistent activity in a group of cells, many working-memory representations unfold in the space of neural activity under the form of continuous trajectories, as neurons successively activate in “relay races” sequences of transient activity (Batuev, 1994; Brody et al., 2003; Cromer et al., 2010; Yang et al., 2014; Schmitt et al., 2017; Enel et al., 2020). In the PFC, neural trajectories can form the substrate for dynamic (Sreenivasan et al., 2014) but also, counterintuitively, for stable representations (Druckmann and Chklovskii, 2012). Neural trajectory-mediated dynamical representations can subserve the retrospective working memory of spatial (Batuev, 1994; Yang et al., 2014) or quantitative (Brody et al., 2003) cues, symbolic categories (Cromer et al., 2010), values (Enel et al., 2020), or behavioral rules (Schmitt et al., 2017). They can also serve prospective working memory in computational processes transforming previously encoded information, such as, for e.g., in visuo-motor transformations (Spaak et al., 2017), in the representation of elapsed time (Tiganj et al., 2017) or in the encoding of forthcoming behaviors (Fujisawa et al., 2008; Ito et al., 2015; Nakajima et al., 2019; Passecker et al., 2019). Neural trajectories in the neural space can also appear as sequences of states that involve combinations of active neurons (Batuev, 1994; Abeles et al., 1995; Seidemann et al., 1996; La Camera et al., 2019). Thus, neural trajectories appear in diverse forms and in different functional contexts where they can map actual trajectories in external spaces, remember previously encountered trajectories, or predict forthcoming trajectories during active computational processes requiring dynamical representations.

Neural trajectories in the PFC are adaptive (Euston et al., 2012; Mante et al., 2013): they are learned and memorized, to be “replayed” later. The timescale of the replay depends on the behavioral context. Regular timescale replays operate at the behavioral timescale, lasting seconds (Batuev, 1994; Fujisawa et al., 2008; Cromer et al., 2010; Mante et al., 2013; Yang et al., 2014; Ito et al., 2015; Schmitt et al., 2017; Tiganj et al., 2017; Nakajima et al., 2019; Passecker et al., 2019; Enel et al., 2020). Thus, such replays unfold online as current behavior is executed in interaction with the external world, to subserve retrospective working memory of past information, on-going dynamical computations, or prospective representation of forthcoming behaviors. Typically, regular replays are triggered by behaviorally–relevant external events (e.g., cues or go signals in working memory tasks, or the current position in navigational tasks). Some replays that may appear as spontaneous can be presumably triggered by internal self-paced decision signals within the PFC (e.g., choices). In all cases, such triggered regular replays rely on internal mechanisms within PFC circuits allowing for the autonomous propagation of proper sequences of activity, once initial neurons of the neural trajectory have been triggered. A major goal of the present study is to decipher how plastic processes allow PFC circuits to learn and replay trajectories, i.e., autonomously generate neural trajectory completion, based on an initial trigger.

Besides, fast timescale replays exist that last a few hundred milliseconds during awake (Jadhav et al., 2016; Mashhoori et al., 2018; Yu et al., 2018; Shin et al., 2019; Kaefer et al., 2020) and sleeping (Euston et al., 2007; Peyrache et al., 2009) states. Beyond their much shorter duration, PFC fast replays are distinct from regular ones, in that they typically operate offline and often co-occur with fast replays in the hippocampal CA1 field (Jadhav et al., 2016). Replay activity in PFC and CA1 presents high degrees of task-dependent spatial and temporal correlations (Jadhav et al., 2016; Yu et al., 2018; Shin et al., 2019), subserving functional coordination combining metric (hippocampus) and task-related (PFC) spatial representations (Pfeiffer and Foster, 2013; Zielinski et al., 2019). These fast replays occur during sharp-wave ripples (SWR) episodes (Jadhav et al., 2016; Yu et al., 2018; Shin et al., 2019), which represent critical events for behavioral learning (Jadhav et al., 2012) and during which animals forge forthcoming decisions (choices, trajectories, for e.g., Jadhav et al., 2016; Mashhoori et al., 2018; Kaefer et al., 2020), based on the recall of past experiences (actions, trajectories, outcomes, for e.g., Jadhav et al., 2012; Mashhoori et al., 2018). Such coordination across both structures presumably emerges through their reciprocal, direct and indirect, synaptic interactions (Witter and Amaral, 2004). Different studies have pointed out information flow biases from CA1 to PFC (Jadhav et al., 2016) or from PFC to CA1 (Ito et al., 2015) directions, depending on behavioral contexts. However, SWR-related replays in the hippocampus correlate with fast replays in reduced subsets of PFC neurons (Jadhav et al., 2016; Yu et al., 2018) that carry generalized spatial representations but not specific trajectories (Yu et al., 2018). Moreover, fast timescale PFC replays are independent of hippocampal replays during computational processes inherent to the PFC, such as rule switching tasks (Kaefer et al., 2020). Therefore, as for regular replays, we examined how plastic processes allow for the emergence of fast timescale replays autonomously within local recurrent PFC circuits.

Neuronal trajectories consist of robust forms of ordered local activity occurring within a disordered global activity, i.e., the chaotic, asynchronous irregular (AI) state characteristic of the prefrontal cortex in the waking state (Destexhe et al., 2003; London et al., 2010). This coexistence poses a problem at the plasticity level, because the noisy AI regime constitutes a potential source of perturbation for synaptic engrams (Boustani et al., 2012; Litwin-Kumar and Doiron, 2014), whereas strengthened connectivity pathways may exert a synchronizing influence on the network, dramatically altering the chaotic nature of background activity. However, there is currently no biophysically-grounded theoretical framework accounting for the way neural trajectories are learned, memorized and replayed within recurrent cortical networks. In principle, synaptic plasticity, a major substrate of learning, may sculpt oriented connective pathways promoting the propagation of neuronal trajectories, because modifications of synaptic connections are activity-dependent. Specifically, the sequential activation of differentially tuned neurons during successively crossed spatial positions (during navigational trajectories) or representational states (during dynamical cognitive processes) could strengthen connections between neurons, creating oriented pathways (referred to as trajectory engrams hereafter) within recurrent cortical networks. If sufficiently strengthened, engrams could allow the propagation of packets of neuronal activity along them. From an initial stimulation of neurons located at the beginning of the engram, due to the strong connections linking them in the direction of the trajectory, neurons could reactivate sequentially, i.e., perform trajectory replay.

Recurrent neural network models have shown that activity-dependent synaptic plasticity rules can enable the formation of trajectory engrams due to long-term potentiation (LTP) and depression (LTD) together with homeostatic scaling (Liu and Buonomano, 2009; Clopath et al., 2010; Fiete et al., 2010; Klampfl and Maass, 2013). Moreover, trajectory engrams can propagate neuronal trajectories through sequential activation of neurons in recurrent model networks (Liu and Buonomano, 2009; Fiete et al., 2010; Klampfl and Maass, 2013; Laje and Buonomano, 2013; Chenkov et al., 2017). However, the above models of neural trajectories do not elucidate the biological basis of learning and replay in neurophysiological situations encountered by PFC networks for several reasons. First, in these models, trajectory learning is either ignored (hard-written trajectory engram; Chenkov et al., 2017), unrelated to behavior (random formation of arbitrary trajectory; Liu and Buonomano, 2009; Fiete et al., 2010), based on artificial learning rules (Laje and Buonomano, 2013) or on biophysically unrealistic rules in terms of neuronal activity and synaptic plasticity constraints (Liu and Buonomano, 2009; Fiete et al., 2010; Klampfl and Maass, 2013). Moreover, trajectory replay is absent (Clopath et al., 2010) or unable to operate from an initial trigger (Klampfl and Maass, 2013), or the ability to memorize and replay trajectory engrams and replays long-term is not tested (Liu and Buonomano, 2009; Clopath et al., 2010; Fiete et al., 2010; Klampfl and Maass, 2013; Laje and Buonomano, 2013; Chenkov et al., 2017). Finally, none of these models evaluate the capacity for trajectory learning and replay in the realistic context where network activity undergoes AI dynamics, whereas it is characteristic of the awake state in the cortex (Destexhe et al., 2003; London et al., 2010). The interactions between synaptic plasticity and AI dynamics has so far only been assessed for static Hebbian engrams (Morrison et al., 2007; Boustani et al., 2012; Litwin-Kumar and Doiron, 2014) but not for dynamic trajectories.

The disordered activity of AI cortical dynamics represents a potentially important source of disturbance at many stages. Indeed, AI regime activity may spontaneously engage plastic processes (before any trajectory presentation), affecting the synaptic network matrix, and leading to altered network dynamics with divergence toward silence or saturation (Siri et al., 2007). Noisy activity may also interfere with the learning of the trajectory engram, by adding erratic entries of calcium to trajectory presentation-induced calcium, leading to jeopardized downstream decoding of calcium as well as erratic switches between long-term potentiation (LTP) and long-term depression (LTD) of synaptic weights. After learning, the continuous effects of AI regime activity-induced plastic processes (LTD or scaling) might erase the trajectory engram during memorization and jeopardize trajectory replay through the destabilizing influence of activity noise. On the other side of the interaction, trajectory learning through Hebbian synaptic plasticity may potentially, in turn, seriously disrupt AI regime activity (Morrison et al., 2007; Siri et al., 2007). Therefore, it remains uncertain whether realistic biological synaptic plasticity rules are well-suited for proper learning and memorizing of trajectory engrams as well as replay of learned trajectories in PFC physiological conditions.

Here, we assessed how learning, memorization and replay of trajectories can arise from biologically realistic synaptic learning rules in physiological PFC networks displaying disordered AI regime activity. To do so, we built a local recurrent biophysical network model designed to capture replay events like those observed in the PFC. Although designed to fit PFC collective spontaneous and triggered neural dynamics, its intrinsic, synaptic and architectural properties are shared across other cortices, allowing for generalization of the results to other non-PFC cortical areas displaying replays. The model displayed AI dynamics and was endowed with realistic Hebbian (Hebb, 1949) spike timing-dependent plasticity (STDP) of excitatory synapses (Bi and Poo, 1998). Synaptic modifications operate through calcium-signaling dynamics capturing NMDA-dependent non-linear pre- to post-synaptic associativity (Graupner and Brunel, 2012) and calcium-dependent phosphorylation of synaptic weights with realistic activity-dependent kinase/phosphatase (aKP) dynamics, conferring a rapid, graded and bidirectional induction together with slow maintenance, consistent with learning and memory timescales observed in animal and human (Delord et al., 2007). Moreover, the model incorporates synaptic scaling, which ensures normalization of pre-synaptic weights, as found in the cortex (Turrigiano et al., 1998; Wang and Gao, 2012; Sweatt, 2016). We show, that, in this realistic model, presenting a stimulus trajectory allowed for rapid learning of a trajectory engram as well as long-term memorization of the trajectory engram despite the disturbing influence of the AI regime. In turn, the STDP learning rule and trajectory engram did not affect the spontaneous AI regime despite their influence on all excitatory neurons from the network. Moreover, we show that trajectory replay accounted for essential aspects of information coding in the PFC, including robustness of replays at the timescale of seconds, fast and regular replays, chunking, large inter-trial variability, and the ability to account for the dual dynamical and persistent aspects of working memory representations.

## Materials and Methods

### Model of Biophysical Local Recurrent Neural Network

We built a biophysical model of a prefrontal local recurrent neural network, endowed with detailed biological properties of its neurons and connections. While the model is presented as PFC, its synaptic and neural properties are generally preserved across cortical areas, allowing for generalization of the results to non-PFC cortical areas. The network model contained *N* neurons that were either excitatory (E) or inhibitory (I) (neurons projecting only glutamate or GABA, respectively; Dale, 1935), with probabilities *p*_*E*_ and *p*_*I*_ = 1−*p*_*E*_, respectively, and $\frac{p_{E}}{p_{I}}=4$ (Beaulieu et al., 1992). Connectivity was sparse (i.e., only a fraction of all possible connections exists, see *p*_*E*→*E*_, *p*_*E*→*I*_, *p*_*I*→*E*_, *p*_*I*→*I*_ parameter values; Thomson, 2002) with no autapses (self-connections) and EE connections (from E to E neurons) drawn to insure the over-representation of bidirectional connections in cortical networks (four times more than randomly drawn according to a Bernoulli scheme; Song et al., 2005; Wang et al., 2006). The synaptic weights *w*_(*i,j*)_ of existing connections were drawn identically and independently from a log-normal distribution of parameters μ_*w*_ and σ_*w*_ (Song et al., 2005).

To cope with simulation times required for the massive explorations ran in the parameter space, neurons were modeled as leaky integrate-and-fire (LIF) neurons. The membrane potential of neuron *j* followed

$$\begin{array}{l} \{\begin{array}{c} C\frac{dV_{\left(j\right)}}{dt}=-(I_{L(j)}+I_{Syn.Rec(j)}+I_{Syn.FF(j)})\\ V_{\left(j\right)}>\theta \to V_{\left(j\right)}=V_{rest} \end{array} \end{array}$$

where neurons spike when the membrane potential reaches the threshold θ, and repolarization to *V*_*rest*_ occurred after a refractory period Δ*t*_*AP*_.

The leak current followed

$$\begin{array}{l} I_{L\left(j\right)}={\bar{g}}_{L}\;\left(V_{\left(j\right)}-V_{L}\right)\; \end{array}$$

where ${\bar{g}}_{L}$ is the maximal conductance and *V*_*L*_ the equilibrium potential of the leak current.

The recurrent synaptic current on post-synaptic neuron *j*, from—either excitatory or inhibitory—pre-synaptic neurons (indexed by *i*), was

$$\begin{array}{l} I_{Syn.Rec(j)}=\sum_{i}^{\;}(I_{AMPA\left(i,j\right)}+I_{NMDA\left(i,j\right)}+I_{GABA_{A}\left(i,j\right)}\\ \;+I_{GABA_{B}\left(i,j\right)})\; \end{array}$$

The delay for synaptic conduction and transmission, Δ*t*_*syn*_, was considered uniform across the network (Brunel and Wang, 2001). Synaptic recurrent currents followed

$$\begin{array}{l} I_{x\left(i,j\right)}={\bar{g}}_{x}\;w_{\left(i,j\right)}\;p_{x\left(i\right)}\left(V_{\left(j\right)}-V_{x}\right)\; \end{array}$$

where *w*_(*i,j*)_ is the synaptic weight, *p*_*x*(*i*)_ the opening probability of channel-receptors and *V*_*x*_ the reversal potential of the current. The NMDA current followed

$$\begin{array}{l} I_{NMDA\left(i,j\right)}={\bar{g}}_{NMDA}\;w_{\left(i,j\right)}\\ \;p_{NMDA\left(i\right)}\;x_{NMDA}\left(V_{\left(j\right)}\right)\left(V_{\left(j\right)}-V_{NMDA}\right)\; \end{array}$$

incorporating the magnesium block voltage-dependence modeled (Jahr and Stevens, 1990) as

$$\begin{array}{l} x_{NMDA}\left(V\right)={\left(1+\left[Mg^{2+}\right]e^{-0.062\;V}/3.57\right)}^{-1}\; \end{array}$$

The channel rise times were approximated as instantaneous (Brunel and Wang, 2001) and bounded, with first-order decay

$$\begin{array}{l} \frac{dp_{x\left(i\right)}}{dt}=-\frac{p_{x\left(i\right)}}{\tau_{x}}+p_{x}\;\left(1-p_{x\left(i\right)}\right)\;\delta \left(t-t_{\left(i\right)}\right)\; \end{array}$$

where δ is the dirac function and *t*_(*i*)_ the times of the pre-synaptic action potentials (APs).

Recurrent excitatory and inhibitory currents were balanced in each post-synaptic neuron (Shu et al., 2003; Haider et al., 2006; Xue et al., 2014), according to driving forces and excitation/inhibition weight ratio, through

$$\begin{array}{l} \{\begin{array}{c} {\bar{g}}_{GABA_{A}}=g_{GABA_{A}}\;\frac{-\left(V_{mean}-V_{AMPA}\right)}{\left(V_{mean}-V_{GABA_{A}}\right)}\;\frac{\sum_{i\in Exc}w_{\left(i,j\right)}}{\sum_{i\in Inh}w_{\left(i,j\right)}}\;\\ {\bar{g}}_{GABA_{B}}=g_{GABA_{B}}\;\frac{-\left(V_{mean}-V_{AMPA}\right)}{\left(V_{mean}-V_{GABA_{B}}\right)}\;\frac{\sum_{i\in Exc}w_{\left(i,j\right)}}{\sum_{i\in Inh}w_{\left(i,j\right)}} \end{array} \end{array}$$

with $V_{mean}=\frac{\left(\theta +V_{rest}\right)}{2}$ being an approximation of the average membrane potential.

Furthermore, all recurrent maximal conductances were multiplied by *g*_*Rec*_, and by *g*_*E*→*E*_, *g*_*E*→*I*_, *g*_*I*→*E*_ or *g*_*I*→*I*_ according to the excitatory or inhibitory nature of pre- and post-synaptic populations.

The feed-forward synaptic current *I*_*Syn*.*FF*(*j*)_ (putatively arising from sub-cortical and cortical inputs) consisted of an AMPA component.

$$\begin{array}{l} I_{Syn.FF\left(j\right)}={\bar{g}}_{AMPA}\;p_{AMPA.FF}\;\left(V_{\left(j\right)}-V_{AMPA}\right)\; \end{array}$$

with a constant opening probability *p*_*AMPA*.*FF*_.

### Synaptic Spike Timing-Dependent Plasticity (STDP)

We used a biophysical model of spike timing-dependent plasticity of excitatory synapses of the network. This rule operated constantly on the weights of the excitatory synapses during simulations. Synaptic weights evolved according to a first-order dynamic (Shouval et al., 2002; Delord et al., 2007) under the control of intra-synaptic calcium (Graupner and Brunel, 2012) through

$$\begin{array}{l} {\dot{w}}_{\left(i,j\right)}\left(t\right)=K_{\max}\frac{Ca{\left(t\right)}^{nH}}{{K_{Ca}}^{nH}+Ca{\left(t\right)}^{nH}}\\ -P_{\max}\frac{Ca{\left(t\right)}^{nH}}{{P_{Ca}}^{nH}+Ca{\left(t\right)}^{nH}}w_{ij}\; \end{array}$$

where the plastic modifications of the synapses, i.e., the phosphorylation and dephosphorylation processes of the synaptic receptor channels, depended on a kinase (e.g., PKC type) and a phosphatase (e.g., calcineurin type) whose allosteric activation was dependent on calcium. Here, *K*_max_ represents the maximum reaction rate of the kinase, *P*_max_ that of the phosphatase, *K*_*Ca*_ and *P*_*Ca*_ the calcium half-activation concentration, *Ca* the synaptic calcium concentration and *nH* is the Hill's coefficient. The term t-LTP, kinase-related, was independent of synaptic weight (“additive” t-LTP) while t-LTD, phosphatase-related, was weight-proportional (“multiplicative” t-LTD), consistent with the literature (Bi and Poo, 1998; van Rossum et al., 2000). This model of STDP is extremely simple, but a detailed implementation would be prohibitive in an RNN of the order of a thousand neurons. There was no term related to the auto-phosphorylation of CaMKII present in many models to implement a form of molecular memory, because on one hand it is not actually involved in the maintenance of memory of synaptic modifications (Chen et al., 2001), and on the other hand memory is ensured here by the dynamics of kinase and phosphatase at low calcium concentration (Delord et al., 2007).

The time dependence of the APs (Bi and Poo, 1998; He et al., 2015) came from calcium dynamics, according to the model of Graupner and Brunel (2012). In this model, synaptic calcium followed

$$\begin{array}{l} Ca\left(t\right)=Ca_{0}+Ca_{pre}\left(t\right)+Ca_{post}\left(t\right)\; \end{array}$$

where the total calcium concentration takes into account pre- and post-synaptic calcium contributions.

Pre-synaptic spiking mediated calcium dynamics followed

$$\begin{array}{l} {\overset{∙}{Ca}}_{pre}\left(t\right)=-\frac{Ca_{pre}\left(t\right)}{\tau_{Ca}}+\Delta Ca_{pre}\underset{i}{\sum }\delta \left(t-t_{\left(i\right)}-D\right)\; \end{array}$$

where the first term corresponds to calcium extrusion/buffering with time constant τ_*Ca*_ and the second term to voltage-dependent calcium channels (VDCC)-mediated calcium entry due to pre-synaptic spiking, with *Ca*_*pre*_ the amplitude of calcium entering at each AP of the presynaptic neuron, *t*_(*i*)_ the times of the pre-synaptic APs, and *D* a delay modeling the time required for the activation of AMPA channels, the depolarizing rise of the associated excitatory post-synaptic potential (EPSP) and the subsequent opening of VDCC that induces this calcium entry.

Post-synaptic spiking-mediated calcium dynamics evolved according to

$$\begin{array}{l} {\overset{∙}{Ca}}_{post}\left(t\right)=-\frac{Ca_{post}\left(t\right)}{\tau_{Ca}}+\Delta Ca_{post}\underset{j}{\sum }\delta \left(t-t_{\left(j\right)}\right)\\ \;+\xi_{PrePost}\underset{j}{\sum }\delta \left(t-t_{\left(j\right)}\right)Ca_{pre}\left(t\right) \end{array}$$

and modeled extrusion/buffering (first-term) as well as calcium entries due to post-synaptic, back-propagated spiking from the post-synaptic soma along the dendritic tree to the synapse, opening VDCC (central term) and NMDA channels (right term). ξ_*PrePost*_ is an interaction coefficient and *t*_(*j*)_ corresponds to the AP time of the post-synaptic neuron. NMDA activation is non-linear and depends on the product of a pre- and a post-synaptic term, representing the dependence of NMDA channel openings on the associative conjunction of pre-synaptic glutamate and post-synaptic depolarization, which releases the magnesium blockade of NMDA channels.

### Synaptic Scaling

Synaptic weights were subjected to a homeostatic form of synaptic normalization, present in the cortex (Turrigiano et al., 1998; Wang and Gao, 2012; Sweatt, 2016), which was modeled in a simplified, multiplicative and instantaneous form (Zenke et al., 2013), following at each time step

$$\begin{array}{l} w_{\left(ij\right)}\left(t+dt\right)=w_{\left(ij\right)}\left(t\right)\frac{\underset{i}{\sum }w_{ij}\left(t=0\right)}{\underset{i}{\sum }w_{ij}\left(t\right)}\; \end{array}$$

This procedure ensured that the sum of the incoming weights on a post-synaptic neuron remained constant despite the plastic modifications due to STDP.

### Estimation of the Time Constant of STDP With Synaptic Scaling

Without synaptic scaling, ẇ_*ij*_ = ẇ_*STDP*_ = *K*(*Ca*)−*P*(*Ca*)*w*. However, synaptic scaling plays an important role in the slow decay of weights, so to study the time constant of this decay we needed to incorporate the effect of synaptic scaling. Considering *n* weights of average value μ_*w*_ incoming upon a post-synaptic neuron, where a proportion *p* of weights undergo STDP of value ẇ_*STDP*_ at time step *t* followed by scaling, then for a given weight *w* within the proportion *p*,

$$\begin{array}{l} w\left(t+\Delta t\right)=\left(w\left(t\right)+{\dot{w}}_{STDP}\Delta t\right)\left(\frac{n\mu_{w}}{n\mu_{w}+np{\dot{w}}_{STDP}\Delta t}\right)\; \end{array}$$

so that after algebra, one obtains

$$\begin{array}{l} \frac{w\left(t+\Delta t\right)-w\left(t\right)}{\Delta t}=\left(1-p\frac{w\left(t\right)+{\dot{w}}_{STDP}\Delta t}{\mu_{w}+p{\dot{w}}_{STDP}\Delta t}\right){\dot{w}}_{STDP}\; \end{array}$$

Passing to the limit Δ*t* → 0, one finds:

$$\begin{array}{l} \dot{w}=\left(1-p\frac{w}{\mu_{w}}\right){\dot{w}}_{STDP}\; \end{array}$$

i.e.

$$\begin{array}{l} \dot{w}=\left(1-p\frac{w}{\mu_{w}}\right)\left(K\left(Ca\right)-P\left(Ca\right)w\right)\; \end{array}$$

To find an estimate of the time constant of plasticity, linearization aroundμ_*w*_ gives

$$\begin{array}{l} \dot{w}\sim \left(P\left(Ca\right)\left(2p-1\right)-\frac{K\left(Ca\right)p}{\mu_{w}}\right)w+K\left(Ca\right)-pP\left(Ca\right)\mu_{w}\; \end{array}$$

so that

$$\begin{array}{l} \tau \sim \frac{\mu_{w}}{\left|pK\left(Ca\right)-\left(2p-1\right)P\left(Ca\right)\mu_{w}\right|}\; \end{array}$$

### Theoretical Dependences Under Asynchronous Irregular Dynamics

The steady-state theoretical concentration of calcium in individual synapses was obtained from fixed-points of *Ca*_*Pre*_ and *Ca*_*Post*_, which yielded

$$\begin{array}{l} {Ca^{{\;}^{*}}\left(\nu_{Pre},\nu_{Post}\right)\;\sim \;Ca}_{0}+\;\tau_{Ca}({\Delta Ca}_{Pre}\nu_{Pre}\\ +{\Delta Ca}_{Post}\nu_{Post}+\xi_{PrePost}{\Delta Ca}_{Pre}\nu_{Pre}\nu_{Post})\; \end{array}$$

which was used to determine STDP modification rates

$$\begin{array}{l} \dot{w}=K\left(Ca^{{\;}^{*}}\right)-P\left(Ca^{{\;}^{*}}\right)w\; \end{array}$$

and to determine the time constant for plasticity, in the case of the network asynchronous irregular regime at low frequency, where *p* = 1, i.e.

$$\begin{array}{l} \tau \sim \frac{\mu_{w}}{\left|K\left(Ca^{{\;}^{*}}\right)-P\left(Ca^{{\;}^{*}}\right)\mu_{w}\right|}\; \end{array}$$

### Weights Within and Outside the Engram

Initial excitatory weights (before the 1 h simulation) were convolved with a centered normalized Gaussian function (σ = 5 *neurons*). Convolved weights with values above 0.1 (times *p*_*E*→*E*_ = 0.35 to take into account inexistent weights) were considered within the engram, the other weights were considered outside the engram. Both weight populations were kept constant and their evolution was studied across time (see **Figures 6**, **7**).

### Trajectory Replay Detection

In order to detect coherent propagating activity pulse packets along the synaptic pathway, we convolved spiking activity across time and neurons with centered normalized Gaussian functions (σ = 30 *ms* and σ ~ 10 *neurons*). Neurons were considered “active” when at least 40% of the convolved frequencies which include them (>5% of normalized Gaussian function maximum) are above 12.5*Hz*. We considered the emergence of an activity packet when it contained more than 20 neurons.

### Spiking Irregularity

To capture spiking irregularity, we quantified the CV (coefficient of variation), CV2 and Lv (time-local variation) of the inter-spike interval (ISI) distribution of the spiking trains of neurons in the network (Compte, 2003; Shinomoto et al., 2005) according to

$$\begin{array}{l} CV=\frac{\sigma_{ISI}}{<ISI>}\; \end{array}$$

$$\begin{array}{l} CV_{2}=<2\frac{\left|ISI_{k+1}-ISI_{k}\right|}{ISI_{k+1}+ISI_{k}}{>}_{k}\; \end{array}$$

$$\begin{array}{l} Lv=<{3\frac{{\left(ISI_{k}-ISI_{k+1}\right)}^{2}}{{\left(ISI_{k}+ISI_{k+1}\right)}^{2}}>}_{k}\; \end{array}$$

where *CV* = *CV*_2_ = *Lv* = 1 for a homogeneous Poisson spike train and = 0 for a perfectly regular spike train where all *ISI* are equal. CV stands around 1 to 2 *in vivo* (Compte, 2003; Shinomoto et al., 2005), representing the global variability of an entire ISI sequence, but is sensitive to firing rate fluctuations. CV2 and Lv stand around 0.25 to 1.25 and 0 to 2, respectively *in vivo* (Compte, 2003; Shinomoto et al., 2005), evaluating the ISI variability locally in order to be less sensitive to firing rate fluctuations. The CV was calculated on every ISI across neurons, while the CV2 and Lv were calculated for each excitatory neuron and averaged across the whole population.

### Spiking Synchrony

Three measures of synchrony were adopted, a synchrony measure *S* (Golomb et al., 2001), pairwise correlation coefficient averaged over all pairs of excitatory neurons < ρ> (Tchumatchenko et al., 2010), and Fano factor *F*. The first two were calculated on the estimated instantaneous neural frequency *f* (Gaussian convolution of spikes, σ = 30*ms*), while the last was calculated on the population sum of spike counts *s*, following

$$\begin{array}{l} S=\sqrt{\frac{Var\left(<f{>}_{n}\right)}{<Var\left(f_{\left(n\right)}\right){>}_{n}}}\; \end{array}$$

$$\begin{array}{l} <\rho >=\frac{1}{N\left(N-1\right)/2}\underset{i}{\sum }\underset{j>i}{\sum }\frac{cov\left(f_{\left(i\right)},\;f_{\left(j\right)}\right)}{\sqrt{Var\left(f_{\left(i\right)}\right)Var\left(f_{\left(j\right)}\right)}}\; \end{array}$$

$$\begin{array}{l} F=\frac{Var\left(\underset{n}{\sum }s_{n}\right)}{<\underset{n}{\sum }s_{n}{>}_{t}}\; \end{array}$$

These measures equal $S=\frac{1}{\sqrt{n_{E}}}\sim \;0.0455$, < ρ> = 0 and *F* = 1 for perfectly asynchronous network activity, and *S* = < ρ> = 1 while F increases for perfectly synchronous network activity.

### Procedures and Parameters

Models were simulated and explored using custom developed code (MATLAB) and were numerically integrated using the forward Euler method with time-step Δ*t* = 0.5*ms* in network models. Unless indicated in the text, standard parameter values were as following. Concerning the network architecture, *N* = 605 *neurons*, *n*_*E*_ = 484 *neurons*, *n*_*I*_ = 121 *neurons*, *p*_*E*→*E*_ = 0.35, *p*_*E*→*I*_ = 0.2056, *p*_*I*→*E*_ = 0.22, *p*_*I*→*I*_ = 0.25, μ_*w*_ = 0.03, σ_*w*_ = 0.02. Concerning the Integrate-and-Fire neural properties, *C* = 1 μ*F*.*cm*^−2^, θ = −52 *mV*, *V*_*rest*_ = −67 *mV*, Δ*t*_*AP*_ = 3 *ms*. Concerning currents, ${\bar{g}}_{L}=\;0.05\;mS.cm^{-2}$, *V*_*L*_ = −70 *mV*, Δ*t*_*syn*_ = 0.5 *ms*, ${\bar{g}}_{AMPA}=0.23\;mS.cm^{-2}$, ${\bar{g}}_{NMDA}=0.9\;mS.cm^{-2}$, $g_{GABA_{A}}=0.3\;mS.cm^{-2}$, $g_{GABA_{B}}=0.017\;mS.cm^{-2}$, *V*_*AMPA*_ = *V*_*NMDA*_ = 0 *mV*, *V*_*GAB*_*A*__*A*__ = −70 *mV*, *V*_*GAB*_*A*__*B*__ = −90 *mV*, [*Mg*^2+^] = 1.5 *mM*, τ_*AMPA*_ = 2.5 *ms*, τ_*NMDA*_ = 62 *ms*, τ_*GAB*_*A*__*A*__ = 10 *ms*, τ_*GAB*_*A*__*B*__ = 25 *ms*, *p*_*AMPA*_ =_*p*_*NMDA*_ = *pGABA*_*A*__ = *p*_*GAB*_*A*__*B*__ = 0.1, *g*_*Rec*_ = 0.65, *g*_*E*→*E*_ = *g*_*E*→*I*_ = *g*_*I*→*E*_ = 1, *g*_*I*→*I*_ = 0.7, *p*_*AMPA*.*FF*_ ~ 0.0951. Concerning synaptic properties, $K_{\max}=3.10^{-3}\;ms^{-1}$, *K*_*Ca*_ = 3 μ*M*, $P_{\max}=3.10^{-3}\;ms^{-1}$, *P*_*Ca*_ = 2 μ*M, nH* = 4, *Ca*_0_ = 0.1 μ*M*, τ_*Ca*_ = 100 *ms*, Δ*Ca*_*pre*_ = 0.02 μ*M*, *D* = 10 *ms*, Δ*Ca*_*post*_ = 0.02 μ*M*, $\xi_{PrePost}=4\;ms^{-1}$.

## Results

### Predicting Fundamental Plastic Properties of PFC Recurrent Networks

**Figure 1:**
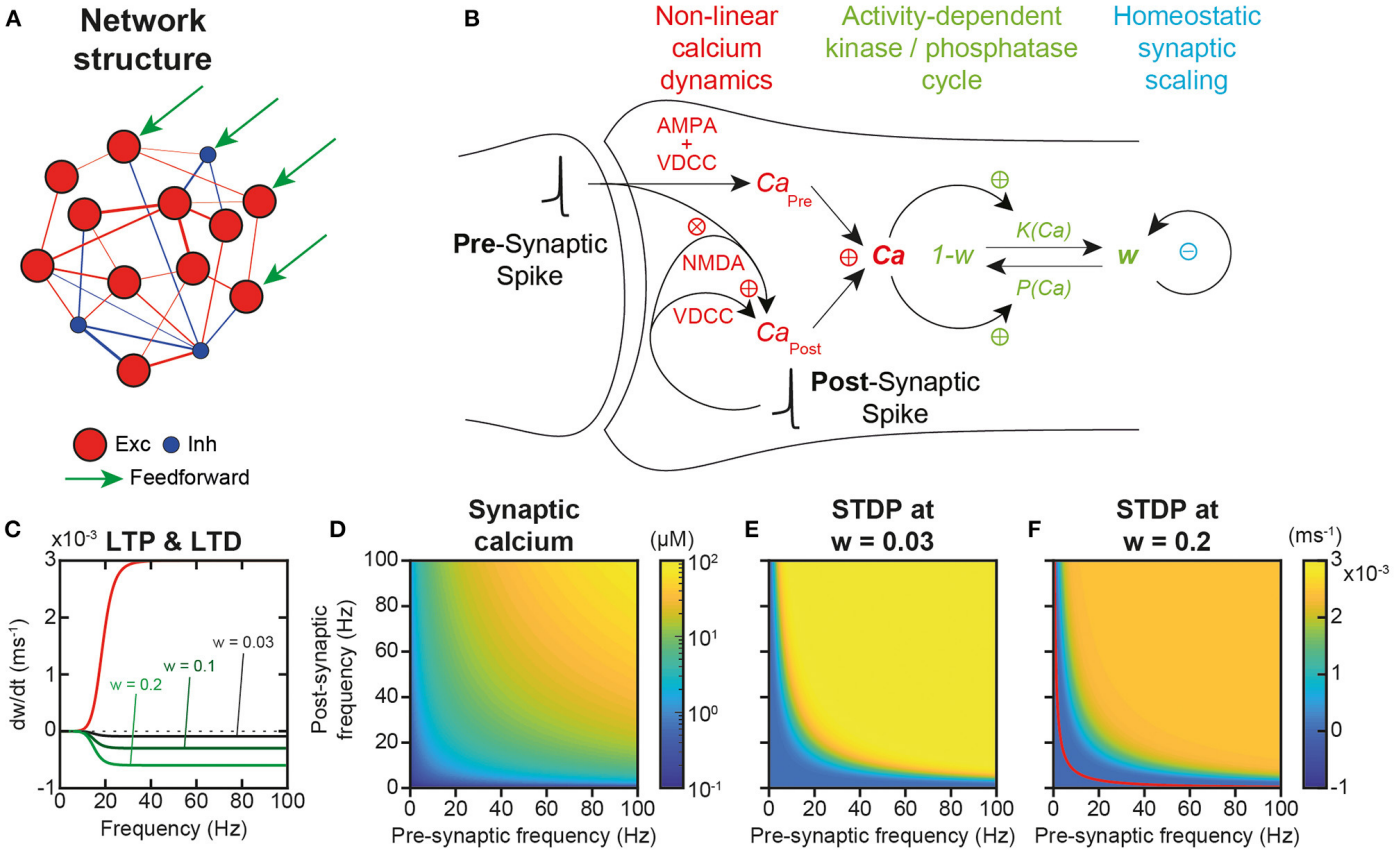
Network structure and plastic properties. **(A)** Scheme of the recurrent network model of the local prefrontal cortex circuit with 484 excitatory (red) and 121 inhibitory (blue) integrate and fire (IAF) neurons. **(B)** Scheme of excitatory synaptic plastic processes. In the post-synaptic compartment, calcium dynamics originates from two distinct sources (*Ca*_*Pre*_ and *Ca*_*Post*_), as well as from extrusion/buffering (Graupner and Brunel, 2012). *Ca*_*Pre*_ arises from pre-synaptic spiking mediated through α-amino-3-hydroxy-5-methyl-4-isoxazolepropionic acid (AMPA) depolarization and the subsequent activation of voltage-dependent calcium (VDCC) channels. *Ca*_*Post*_ models calcium entries due to post-synaptic spiking back-propagated from the post-synaptic soma to the synapse, opening VDCC and N-methyl-D-aspartate (NMDA) channels. NMDA activation is non-linear and depends on the interaction of pre- and post-synaptic spiking to account for the associative dependence of NMDA channel openings on the conjunction of pre-synaptic glutamate and post-synaptic depolarization that releases magnesium blockade. Plastic modifications operate through calcium-dependent phosphorylation and dephosphorylation of channel AMPA receptors that determine the synaptic weight (aKP model; Delord et al., 2007). Synaptic scaling continuously normalizes weights so as to insure the homeostatic regulation of the sum of incoming (pre-synaptic) weights for each individual neuron (Turrigiano et al., 1998). **(C)** Both long-term spike timing-dependent potentiation (t-LTP) and long-term spike timing-dependent depression (t-LTD) increase non-linearly with pre- and post-synaptic spiking frequency (ν_*Pre*_ = ν_*Post*_ = ν), due to the allosteric calcium-activation of both enzymes. Kinase-mediated t-LTP is additive, i.e., independent of synaptic weight, while phosphatase-mediated t-LTD is multiplicative, i.e., weight-proportional (Bi and Poo, 1998; van Rossum et al., 2000). **(D)** Because of the associative dependance of NMDA-mediated calcium entry to pre- and post-spiking, synaptic calcium depends multiplicatively on pre- and post-synaptic spiking frequencies. **(E)** In the spontaneous AI regime, plastic modifications are virtually null because STDP plasticity occurs similarly at all synapses, with synaptic scaling compensating STDP (see Results). **(F)**. In synapses connecting neurons in the engram of a learned trajectory, where plasticity has occurred in a subset of synapses, Hebbian t-LTP dominates at large multiplicative pre-/post- frequencies and Hebbian t-LTD at lower frequencies (separated by the red curve for which plasticity is null, see Results).

To evaluate neural trajectory learning, memorization and replay, we studied a local prefrontal cortex (PFC) recurrent network model, with 484 excitatory and 121 inhibitory integrate and fire (IAF) neurons with topographically tuned feed-forward inputs. Synaptic connections were constrained by cortical connectivity data, following Dale's law, sparseness and log-normal weight distributions, and α-amino-3-hydroxy-5-methyl-4-isoxazolepropionic acid (AMPA) and N-methyl-D-aspartate (NMDA) excitatory and γ-aminobutyric acid (GABA-A and GABA-B) inhibitory synaptic currents (Figure 1A; see *Materials and Methods*). Most synaptic and neural properties, while present in PFC, are generic across cortex, such that the following results can be generalized to non-PFC cortical areas.

Excitatory synapses were plastic, i.e., endowed with realistic calcium dynamics (Graupner and Brunel, 2012) accounting for linear voltage-dependent calcium channels (VDCC)-dependent and non-linear NMDA calcium entries, as well as for linear extrusion and buffering (Figure 1B). These calcium dynamics are responsible for the temporal asymmetry of pre- and post-synaptic spike-timing dependent (STDP) plastic modifications (Bi and Poo, 1998; He et al., 2015). Note, however, that with these realistic calcium dynamics, plasticity essentially depends on firing frequency rather than on the precise timing of spikes, because of the frequency and variability of *in vivo*-like spiking (Graupner et al., 2016).

Plastic modifications operated through calcium-dependent kinase-phosphatase kinetics (Delord et al., 2007), which accounts for their fast induction and slower maintenance dynamics (Figure 1B). No Ca^2+^/calmodulin-dependent protein kinase II (CaMKII) auto-phosphorylation was present because it is actually not involved in the maintenance of synaptic modifications (Chen et al., 2001; Lengyel et al., 2004). Rather, the long-term maintenance of plastic modifications emerges from kinase and phosphatase dynamics at low calcium concentrations (see below; Delord et al., 2007). Besides, synapses underwent synaptic scaling (Figure 1B), which ensures total weight normalization at the neuron level, as observed in the cortex (Turrigiano et al., 1998; Wang and Gao, 2012; Sweatt, 2016) and, as a consequence, introduces competition between synaptic weights within each neuron (intra-neuronal inter-synaptic competition).

Most importantly, plasticity operated online—i.e., permanently, without offline learning periods—on excitatory synaptic weights, as a function of neuronal activity in the network, whether it corresponds to the spontaneous, asynchronous and irregular (AI) activity of the network, the activity evoked by the feed-forward currents during the input presentation of an example trajectory, or the replay activity after learning (see below). Both kinase-mediated long-term spike timing-dependent potentiation (t-LTP) and phosphatase-mediated long-term spike timing-dependent depression (t-LTD) increased non-linearly with pre- and post-synaptic spiking frequency, due to the allosteric activation of enzymes by calcium (Figure 1C). However, they differed in that kinase-mediated t-LTP was independent of synaptic weight (additive or hard-bounded) while phosphatase-mediated t-LTD was weight-proportional (multiplicative or soft-bounded), consistent with the literature (Bi and Poo, 1998; van Rossum et al., 2000; Figure 1C). In the model, the steady-state theoretical concentration of calcium in individual synapses depended multiplicatively upon pre-synaptic and post-synaptic spiking activity (Figure 1D), from which one could compute the rate of STDP as a function of pre- and post-synaptic spiking frequency (Figures 1E,F) see Materials and Methods). In conditions with weak synaptic weights, such as prior to learning, t-LTP dominated at all frequencies because t-LTD is multiplicative and thus scaled by, here, very low synaptic weights. Thus, STDP effects were always positive and depended multiplicatively on pre- and post-synaptic frequencies (Figure 1E). By contrast, when plasticity had previously occurred (*w* = 0.2), such as in the engram of a learned trajectory (see below), t-LTD was stronger due to the stronger weights, and the model predicted Hebbian t-LTP at large multiplicative pre-/post-frequencies and t-LTD at lower frequencies (Figure 1F). In the following, we explore the extent to which these predictions are correct in simulations of the whole network model under spontaneous AI dynamics with synaptic scaling, and when assessing learning and memorization upon trajectory presentation.

### Stability of Network AI Dynamics Under Synaptic Plasticity

**Figure 2:**
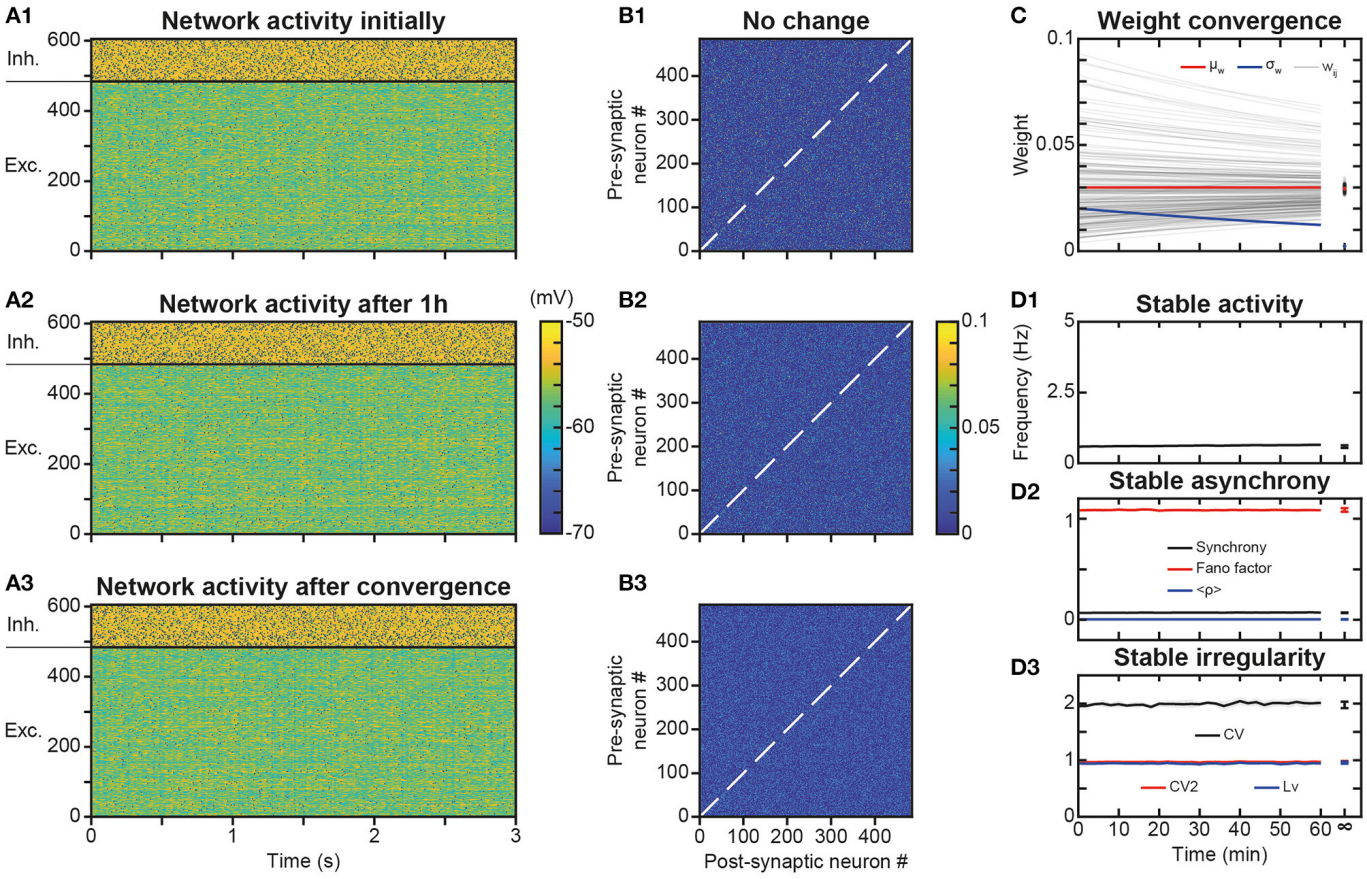
Stability of spontaneous irregular asynchronous (AI) network dynamics under synaptic plasticity. **(A1–A3)** Membrane potential of network neurons during 3 s of spontaneous AI regime in the absence of plasticity **(A1)**, after 1 h of plasticity **(A2)** and after full convergence of synaptic weights due to plasticity **(A3)**. The same initial random connectivity matrix is used for simulations in **(A1–A3)**. Spikes indicated by black dots. Full convergence of the synaptic matrix was obtained by simulating the networks with very fast kinetic constants. **(B1–B3)** Synaptic weights between excitatory neurons of the network at the end of each of simulations presented in **A1–A3**. **(C)** Convergence of synaptic weights toward the mean weight of their post-synaptic neuron as a function of time, due to synaptic scaling normalization (black curves, see Results). Time evolution of the mean (red curve) and standard deviation (blue curve) of synaptic weights. For sake of clarity, only a random selection of synapses is shown. The mean is constant and the standard deviation decreases with time, due to scaling. **(D)**. Average excitatory neural spiking frequency **(D1)** and irregularity **(D3)** and excitatory population synchrony **(D2)** quantifiers, as a function of time, for five different simulations of the network with different realizations of the initial random synaptic matrix. Dots on the right indicate values obtained from network simulations after full convergence of synaptic weights. Shaded areas represent 95% confidence intervals of the mean.

A potential issue of synaptic plasticity in network models remains its sensitivity to spontaneous activity. Hence, before testing the possible role of STDP in trajectory learning and replay, we first studied the effect of STDP on the spontaneous regime, with the aim of verifying that network activity remained stable over the long term and that neurons always discharged in the AI regime. Indeed, Hebbian or post-Hebbian rules of the STDP type, by modifying the matrix of synaptic weights, may lead to saturation of neuronal activity and a collapse of the complexity of the dynamics, from initially AI chaotic activity characteristic of the waking state (Destexhe et al., 2003; London et al., 2010), to activity of the limit-cycle or fixed point type (Siri et al., 2007). We considered here as long term the 1 h time scale, which is the scale classically used experimentally to test the memory of synaptic plasticity modifications (Bi and Poo, 1998). Moreover, a duration of 1 h extends way beyond the classical time scales used in models (Morrison et al., 2007; Boustani et al., 2012; Litwin-Kumar and Doiron, 2014). For this purpose, we have observed the activity (Figure 2A) and connectivity (Figure 2B) of the network at different time scales, in order to reveal possible modifications in the network behavior.

Simulations showed that the spontaneous activity of the network was identical without plasticity (Figure 2A1), after 1 h in the presence of plasticity (Figure 2A2) and after full convergence (Figure 2A3) of weight matrix dynamics. This observation is consistent with the absence of changes in the connectivity matrix in the presence of STDP, even after 1 h of simulation (Figures 2B2,B3), compared to the condition without STDP (Figure 2B1). Mechanistically, the low spiking frequency of neurons resulted in moderate average elevations of calcium above its basal concentration in synapses, so that kinase and phosphatase were only very weakly activated. Therefore, weights underwent extremely slow plastic modifications where additive t-LTP (which dominated the multiplicative t-LTD at weak weights) was compensated by synaptic scaling. Due to these effects, weights converged toward the mean initial weight of their post-synaptic neuron (Figure 2C) with an apparent time constant of 2 h, close to the theoretical estimation of the time constant of plasticity (see *Materials and Methods* and *Discussion*), which predicts a time constant of 1.95 h during learning at low spiking frequencies and calcium concentrations (*Ca*~*Ca*_0_) in the AI regime. These steady-state values were normally distributed, with a constant mean value (due to the synaptic scaling) and a decreasing standard deviation, due to the homogenization of weights within each post-synaptic neuron (Figure 2C). Even with this more homogeneous synaptic matrix (Figure 2B3), AI dynamics were preserved (Figure 2A3). Indeed, excitatory frequency was stable (Figure 2D1), as well as markers of synchrony (Figure 2D2) and irregularity (Figure 2D3). Thus, overall, the activity regime of the network was not altered by the presence of plastic processes. Note that in PFC circuits experiencing dynamically changing feed-forward inputs, convergence of the synaptic matrix may be attenuated or even non-existent.

### Learning Trajectory Engrams Under AI Dynamics

**Figure 3:**
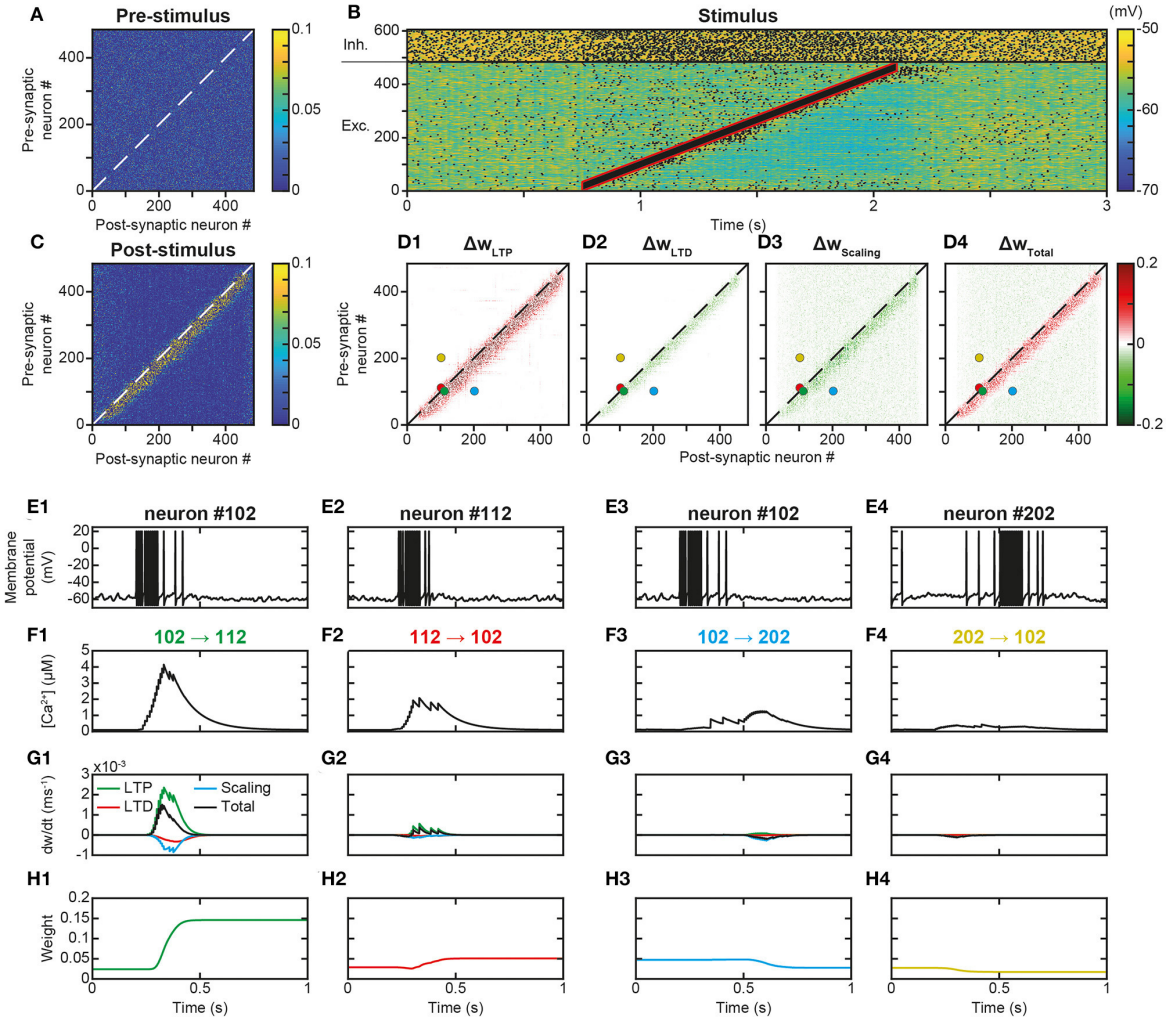
Learning a trajectory stimulus into a trajectory engram. **(A)**. Synaptic matrix between excitatory neurons prior to stimulus presentation. **(B)**. Membrane potential of network neurons in response to the presentation of a trajectory stimulus (stimulus in red) that successively activates all excitatory neurons over a duration of 1,350 ms. Spikes indicated by black dots. **(C)**. Synaptic matrix between excitatory neurons after stimulus presentation. **(D1–D4)**. Weight modifications resulting, after trajectory presentation, from t-LTP **(D1)**, t-LTD **(D2)**, scaling **(D3)**, and their sum **(D4)**. **(E–H)** Membrane potential **(E)**, calcium **(F)**, plastic rates **(G)** and synaptic weight dynamics **(H)** during the passage of the trajectory stimulus in a pair of neurons with nearby topographical tuning #102 **(E1)** and #112 **(E2)** and their reciprocal connections 102 → 112 **(F1-H1)** and 112 → 102 **(F2-H2)**, and in a pair of neurons with more distant topographical tuning #102 **(E3)** and #202 **(E4)** and their reciprocal connections 102 → 202 **(F3-H3)** and 202 → 102 **(F4-H4)**.

Trajectory learning during network activity has already been investigated in the theoretical literature, but either without chaotic dynamics or using biologically unrealistic learning rules (see *Introduction*). To test for the possibility of learning trajectories within physiologically irregular activity, we presented to the network a moving stimulus (Figure 1A, feedforward connections) that successively activated all the excitatory neurons over 1,350 ms (Figure 3B). Such a stimulation corresponds to a displacement speed of ~0.3 neurons/ms, where each excitatory neuron was stimulated for ~100 ms and discharged at ~100 Hz. This single stimulus presentation triggered neural activity much stronger than the spontaneous activity, sufficient to modify the matrix of synaptic weights. Indeed, whereas the synaptic matrix was initially formed of low random weights (Figure 3A), after presentation, the weights of synapses connecting neurons activated by the stimulus at close successive times were increased (Figure 3C). This diagonal band of increased weights formed an oriented connectivity path along stimulus-activated neurons and is referred to as the trajectory engram hereafter. Weight modifications inside and outside this trajectory engram resulted from increases due to t-LTP (Figure 3D1, Δw_LTP_) and decreases due to t-LTD (Figure 3D2, Δw_LTD_). Moreover, the homeostatic process of synaptic scaling, which ensures the constancy of the sum of the incoming weights of the cortical neurons, decreased the total incoming synaptic weights on post-synaptic neurons, in order to compensate for weight modifications due to STDP (Figure 3D3, Δw_Scaling_). In fine, STDP and scaling led together to an increase in engram weights and a slight decrease in off-engram weights (Figure 3D4, Δw_Total_; also observe the darker area in Figure 3C, compared to Figure 3A).

The observation, on a local scale, of the details of the processes at work for the synapses linking the neurons of the engram allowed for a better understanding of these network effects. For illustration, neurons #102 and #112, with close spatial topographical tuning, discharged one following the other with partial overlap during the stimulus (Figure 3E). At the level of the synapse between neurons #102 and #112 (102 → 112), whose orientation was that of the trajectory, the arrival of pre-synaptic action potentials (APs) was followed by that of postsynaptic APs (pre #102 then post #112 neuron, Figures 3E1,E2), which triggered a massive input of calcium via the VDCC channels and the NMDA receptor channels (Figure 3F1). Conversely, in the synapse 112 → 102, for which the sequence of arrival of the APs was reversed (pre #112 then post #102 neuron), NMDA channels did not open (see above), such that the calcium input resulted only from the VDCC channels and was thus moderate (Figure 3F2). These calcium elevations activated the kinases and phosphatases, which, respectively, phosphorylated and dephosphorylated AMPA channels, increasing (t-LTP) and decreasing (t-LTD) synaptic weights (only phosphorylated AMPA channels are functional and ensure synaptic transmission). These kinase and phosphatase activations were important for synapse 102 → 112 (Figure 3G1), but less so for the synapse 112 → 102 (Figure 3G2). For both synapses (Figures 3G1,G2), the phosphatase was more strongly activated (lower half-activation; Delord et al., 2007), but the resulting t-LTD modification rate was low, because it is multiplicative, i.e., it scales with synaptic weight, which was low. Conversely, the rate of modification due to t-LTP was higher because it is additive and depends only on kinase activation (van Rossum et al., 2000). These STDP effects, cumulated with those of scaling, resulted in a positive speed (increase in weight), which was strong for synapse 102 → 112 (Figure 3G1) and very weak for synapse 112 → 102 (Figure 3G2). Together, these plastic processes increased the weight of the synapse oriented in the same direction as the stimulus (Figure 3H1) leaving the weight of the synapse of opposite orientation almost unchanged (Figure 3H2).

For neurons whose receptive fields were more spatially distant, activation by the stimulus occurred at more temporally distant times (for example, neurons #102 and #202, Figures 3E3,E4). In this case, regardless of the sequence of arrival of the APs in both neurons, their succession was too distant in time to open NMDA channels, so that incoming calcium came only from the VDCC channels and was therefore low (Figures 3F3,F4). Consequently, kinase and phosphatase were weakly activated, resulting in virtually null STDP velocity (Figures 3G3,G4). Synaptic scaling (Figures 3G3,G4), induced by the increase of weights in the engram (Figures 3H1,H2), ultimately decreased synaptic weights (Figures 3H3,H4). As such, there was no learning of any trajectory between distant neurons, contrary to what happened between closer neurons.

### Trajectory Replays From Learned Trajectory Engrams

**Figure 4:**
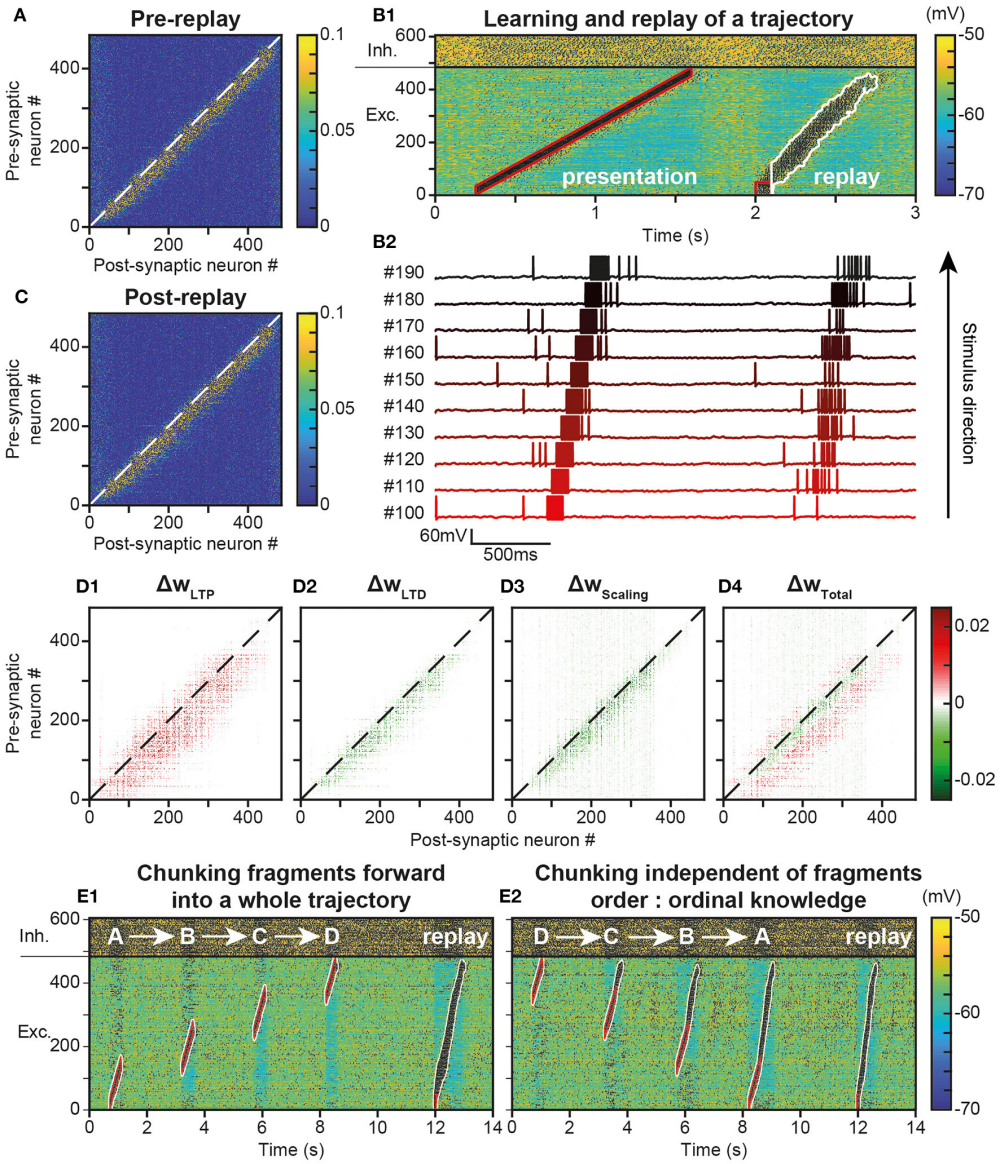
Replay of learned trajectories. **(A)**. Synaptic matrix between excitatory neurons after stimulus presentation but prior to trajectory replay. **(B)**. Membrane potential of network neurons (**B1**, spikes indicated by black dots) in response to the trajectory stimulus, followed by a transient trajectory replay triggered by stimulating the start of the trajectory (neurons #1–50, stimulus in red). Membrane potential of a selected subset of neurons along the trajectory (**B2**, arbitrary colors). **(C)**. Synaptic matrix between excitatory neurons after stimulus and replay. **(D)**. Weight modifications resulting, after compared to before trajectory replay, from t-LTP **(D1)**, t-LTD **(D2)**, scaling **(D3)**, and their sum **(D4)**. **(E)** Recapitulation of the whole trajectory after separately learning four individual trajectory fragments (ABCD) in the forward order (**E1**; chunking) or backward order (**E2**; ordinal knowledge). Each fragment corresponds to 180 neurons. Fragments overlap over 65 neurons.

In behaving animals, learnt trajectories are replayed later in appropriate behavioral conditions. In the model, we assessed whether trajectories could be replayed, the dynamics of trajectory replays and the way they affect the network connectivity compared to before they occur (Figure 4A). Trajectory replay was defined as the reactivation of neurons of the entire trajectory engram, after temporarily stimulating only initial neurons at the beginning of the engram. To assess trajectory replay in the network, we applied a stimulus of 100 ms to the first 50 neurons of the engram, 500 ms after trajectory learning was completed (Figure 4B). We found that the network was able to replay the trajectory entirely after learning (Figure 4B1). Fundamentally, the replay emerged because neurons were linked by strong synapses so that preceding neurons activated subsequent neurons in the engram, forming an oriented propagating wave (Figure 4B2).

Because it activated neurons at several tens of Hz, the replay could have brought into play plastic processes at the synapses forming the engram, and, in doing so, either reinforce or diminish their weights, possibly disturbing or even destroying the engram. To evaluate these possibilities, we observed the variation of synaptic weights before and after the replay. We found that after replay, the engram was still present (Figure 4C) and its structure identical to that before replay (Figure 4A). However, when dissecting the effects at work, we found that the engram had slightly thickened during the trajectory replay, due to the combined effect of t-LTP (Figure 4D1 Δw_LTP_), t-LTD (Figure 4D2 Δw_LTD_) and scaling (Figure 4D3 Δw_Scaling_). Weights above and below the engram increased, whereas weights slightly decreased within the engram (Figure 4D4, Δw_Total_, red fringes).

Up to this point, the neural trajectory was presented as a whole. However, whole trajectories are generally not accessible directly to the PFC. Rather, PFC circuits generally encounter elementary trajectory fragments at separate points in time to produce prospective planning of future behaviors (Ito et al., 2015; Mashhoori et al., 2018; Kaefer et al., 2020), as well as learn transitions between them and chunk fragments together as whole trajectories independently of their presentation order (ordinal knowledge) (Ostlund et al., 2009; Dehaene et al., 2015). We trained the network with four fragments of the whole trajectory, noted A-D, that overlapped at their extremities and which were presented sequentially every 2 s, so as to learn separately different parts of the trajectory (Figure 4E). We found that, once fragments were presented in forward order (ABCD), stimulating neurons at the beginning of the A fragment induced propagation of activity that recapitulated the whole trajectory, by subsequently recalling ABCD fragments in the forward order (Figure 4E1). Therefore, the network was able to learn trajectory fragments themselves and the transitions between fragments so as to chunk them into a whole trajectory. Moreover, we found that chunking was possible even when fragments had been learned in reverse order (DCBA; Figure 4E2). Hence, the network was able to replay a chunked trajectory based on the presentation of overlapping stimuli, independently of their order of presentation.

### Functional Diversity of Trajectory Replays

**Figure 5:**
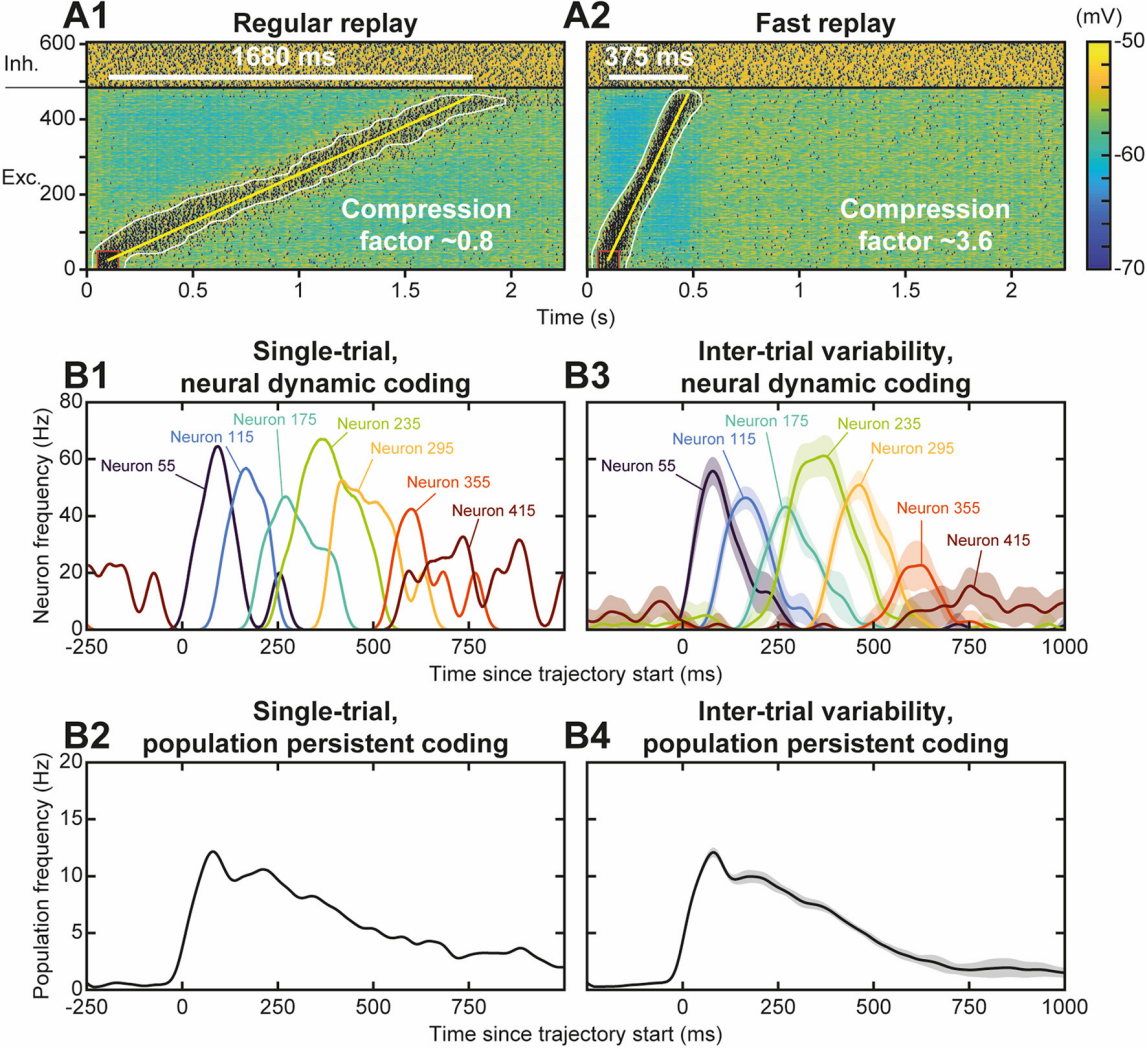
Functional diversity of trajectory replays. **(A)** Trajectory replay duration (upper left white bar) and compression factor (tCF; lower right) depend on the NMDA conductance decay time constant (τ_*NMDA*_, range 30–150 ms). NMDA maximal conductance was scaled (range 0.475–1.8) so as to insure similar levels of firing frequency drive during trajectory replays. Regular **(A1)** and fast **(A2)** timescale replay are due to slower and faster NMDA dynamics. **(B)**. Single-trial **(B1, B2)** and inter-trial variability **(B3,B4)** of firing frequency of individual neurons **(B1,B3)** and of the population **(B2,B4)** for 10 different simulations similar to the replay shown Figure 4B. Lines represent mean values, shaded regions represent 95% confidence intervals of the mean.

Neural activity during the replay was less focused than the stimulus trajectory (Figure 4B), i.e., it involved more (~90 vs. 35) neurons, spiking at a lower (~65 vs. 100 Hz) discharge frequency. The replay also unfolded at a faster speed, lasting ~750 ms—for a stimulus of 1,350 ms—so that it exhibited a temporal compression factor (tCF) of ~1.8, which is situated between fast and regular timescale replays observed in animals. Regular timescale replays operate at the timescale of behaviors they were learnt from, i.e., a few seconds (in navigation or working memory tasks, e.g.), hence typically displaying tCF~1. By contrast, fast timescale replays last several hundred ms in the awake PFC (200–1,500 ms; Jadhav et al., 2016; Mashhoori et al., 2018; Kaefer et al., 2020), yielding several-fold compression factors (tCF ~ 2–15). We assessed whether varying biophysical parameters of the network could account for durations and tCF ranges characterizing regular and fast replays. As regular and fast timescale replays frequently alternate within trials in behavioral tasks, we discarded trivial replay speed control that can be readily obtained by scaling structural parameters that vary at extremely slow timescales (e.g., number of neurons in the trajectory, synaptic delay, etc., not shown). Rather, we focused on synaptic and intrinsic neuronal properties likely to be rapidly regulated by ongoing neuromodulation in the PFC, as attentional demands or reward outcomes vary at the trial timescale. Among passive and synaptic neuronal parameters tested, the NMDA conductance decay time constant (τ_*NMDA*_) emerged as a critical factor controlling the duration and tCF of replays. Hence, the same network, taught with the same trajectory and stimulated with the same initiation stimulus, could generate a large range of replay timescales spanning from regular (duration 1,680 ms, tCF = 0.8; Figure 5A1) to fast (duration 375 ms, CF ~ 3.6; Figure 5A2) replays, when the decay time constant of NMDA, τ_*NMDA*_, was varied. Consistently, dopaminergic neuromodulation, the major determinant of reward signaling, rapidly slows the decaying dynamics of NMDA currents in PFC circuits (Chen et al., 2004; Onn and Wang, 2005; Onn et al., 2006). Such neuromodulatory effects, as well as others forms of neuromodulation of NMDA dynamics (Lutzu and Castillo, 2021) may control the duration and compression factor of trajectory replays, as well as the relative rate of occurrence of regular vs. fast timescale replays. Inspecting neuronal activity during replays in terms of firing frequency, we found that in single replays individual neurons displayed a sequence of overlapping transient bumps of activity of a few hundred milliseconds (Figure 5B1) resembling “relay race” of PFC individual activities during regular replays in working memory tasks (Batuev, 1994; Brody et al., 2003; Cromer et al., 2010; Yang et al., 2014; Schmitt et al., 2017). By contrast, the averaged frequency over the population of excitatory neurons displayed a persistent decaying activity pattern that lasted at the second time scale (Figure 5B2) and mimicked population-level working memory maintenance in the PFC (Murray et al., 2017; Cavanagh et al., 2018; Enel et al., 2020). This dichotomy recalls that found in the PFC, whereby individual neurons encode information at short timescale while the population holds stabilized persistent representations on longer timescales (Meyers et al., 2008; Murray et al., 2017; Cavanagh et al., 2018). Moreover, we found that inter-trial variability for each neuron was important, due to disordered network AI dynamics, and that it increased as activity traveled later in the trajectory in individual neurons (Figure 5B3) and at the population level (Figure 5B4), as found experimentally (Compte, 2003; Shafi et al., 2007; Tiganj et al., 2017).

Globally, the model thus not only indicated that it was possible to learn trajectories online by creating synaptic engrams, thanks to the STDP-type plasticity rule. It also showed that learned trajectories were functional as a memory process, in the sense that their replay was possible and globally preserved the synaptic structure of the learned engram. Finally, the model accounted for the large functional diversity of replays observed in behaving animals, both with regard to the timescale (fast vs. regular) they exhibit, as well as to the type of coding (dynamical vs. stable) they may subserve in navigational or working memory tasks.

### Stability of Network AI Dynamics in the Presence of Trajectory Engrams

**Figure 6:**
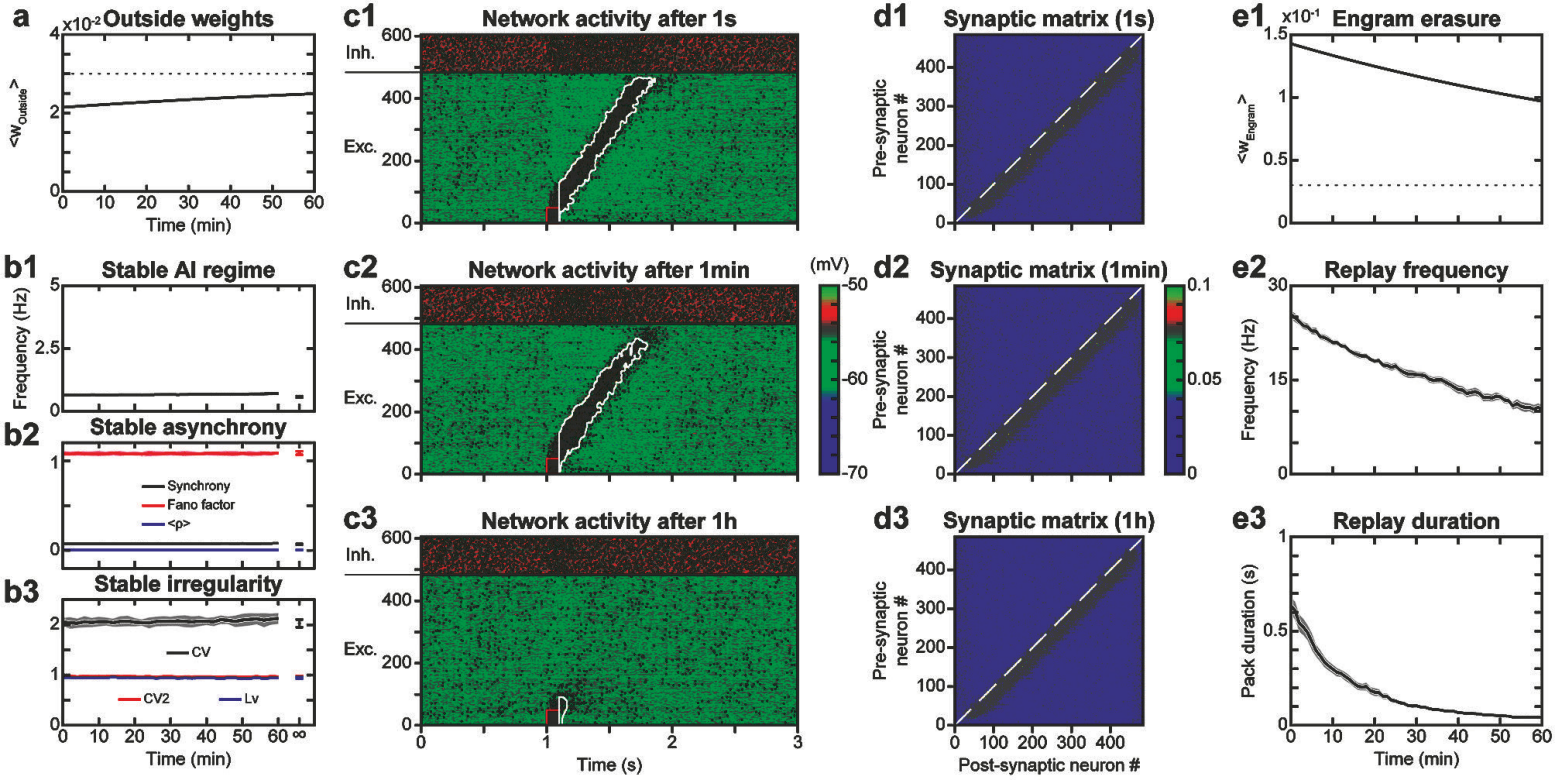
Stability of the spontaneous AI regime in the presence of the engram. **(A)**. Average synaptic weights outside the engram after learning for 1 h. Shaded areas represent 95% confidence intervals of the mean for 5 network simulations. **(B)**. Networks dynamics after learning for 1 h: frequency **(B1)**, synchrony **(B2)**, and irregularity **(B3)** of excitatory neurons. Shaded areas as in **(A)**. **(C)**. Membrane potential of neurons in the neural network for 3 s following a replay stimulation of the 50 first neurons at 1 s **(C1)**, 1 min **(C2)** or 1 h **(C3)** after trajectory learning. **(D)** Synaptic matrices between excitatory neurons of the network, at the end of the simulations presented in **(C)**. **(E)** Network engram synaptic weight average **(E1)** as well as frequency **(E2)** and duration **(E3)** of trajectory replays during 1 h after trajectory learning. Shaded areas as in **(A)**.

After evaluating the stability of the learned trajectory in the presence of AI network activity, we asked the symmetrical question, i.e., whether the engram of a previously learned trajectory could alter the irregular features of spontaneous network dynamics. Indeed, the altered synaptic structure (which implies large weights in all neurons of the recurrent network) may induce correlated activations of neurons (e.g., partial replays) resulting in runaway activity-plasticity interactions and drifts in network activity and synaptic structure. We monitored network connectivity (Figure 6A) and activity dynamics (Figures 6B1–B3) for 1 h to assess the stability of the spontaneous AI regime in the presence of the engram. We observed that following learning of the engram, synaptic weights outside the engram (i.e., responsible for the AI dynamics) increased exponentially toward their new steady-state in a very slow manner (Figure 6A) with an apparent time constant of 1.91 h, consistent with the theoretical estimation of 1.95 h (see above). This increase resulted from the decrease of within-engram large synaptic weights via synaptic scaling (Figure 6E1, see above). Despite this slow and moderate structural reorganization, AI dynamics were preserved with stable frequency (Figure 6B1), synchrony (Figure 6B2), and irregularity (Figure 6B3). Thus, overall, both the synaptic structure outside the engram as well as the spontaneous AI regime remained stable in the presence of the engram.

### Memory of Trajectory Engrams in the Presence of Network AI Dynamics

We then studied whether the spontaneous AI activity could disrupt the engram of the learned trajectory and the possibility for trajectory replay. Indeed, the trajectory engram may be gradually erased, due to AI activity at low frequency favoring t-LTD, or even amplified, due to the activity in the trajectory engram caused by plasticity (resulting in further plasticity runaway). To do so, we assessed the timescale of potential drifts in engram connectivity and activity following learning, and of the network ability to replay the engram. Intuitively, engram erasure, runaway or stability probably depended on network dynamics after learning: spontaneous AI regime, spontaneous replays, or other forms of activity.

To address these questions, we simulated the network for 1 h after trajectory learning and recorded “snapshots” of the continuous evolution of the synaptic matrix every minute. Using these successive recorded matrices as initial conditions for independent simulations of replays, we were able to quantify network ability for trajectory replay, at different times of the evolution of the network. We found that while trajectory replay occurred in full after 1 s, activating all neurons of the trajectory (Figure 6C1), it was slightly attenuated after 1 min (last neurons spiking at lower frequency; Figure 6C2) and failed after 1 h (Figure 6C3). Observing the synaptic matrix at these three moments allowed us to understand the origin of this degradation in replay ability. Indeed, whereas after 1 min (Figure 6D2), the synaptic weights of the engram changed only a little compared to 1 s (Figure 6D1), the engram was narrowed and weights attenuated after 1 h (Figure 6D3). Such degradation of the engram was probably the cause of the failure to replay the trajectory 1 h after learning.

To more precisely monitor degradation of the trajectory engram and replay, we measured averaged engram weights as well as replay frequency and duration across time. We found that the engram weights declined exponentially with a fitted time constant of 1.91 h (Figure 6E1), very close to that predicted by the theory (1.95 h). The measures of trajectory replay decreased faster than the engram weights, with time constants of ~54 min for mean frequency during the replay (Figure 6E2) and ~13 min for replay duration (Figure 6E3). Specifically, replay of the full trajectory lasted 4 min. The degradation of trajectory replay was mainly due to progressive replay failure in the neurons located later in the trajectory engram. The faster decrease in trajectory activity, compared to the average engram weights, was probably a consequence of a cooperative mechanism of propagation in the engram: the non-linearity in NMDA current activation, requiring synergistic activation of pre- and post-synaptic neurons in the engram, rendered the propagation of activity non-linearly sensitive to decreases in engram weights.

### Repeated Trajectory Replays Can Destabilize Trajectory Engrams and Replays

**Figure 7:**
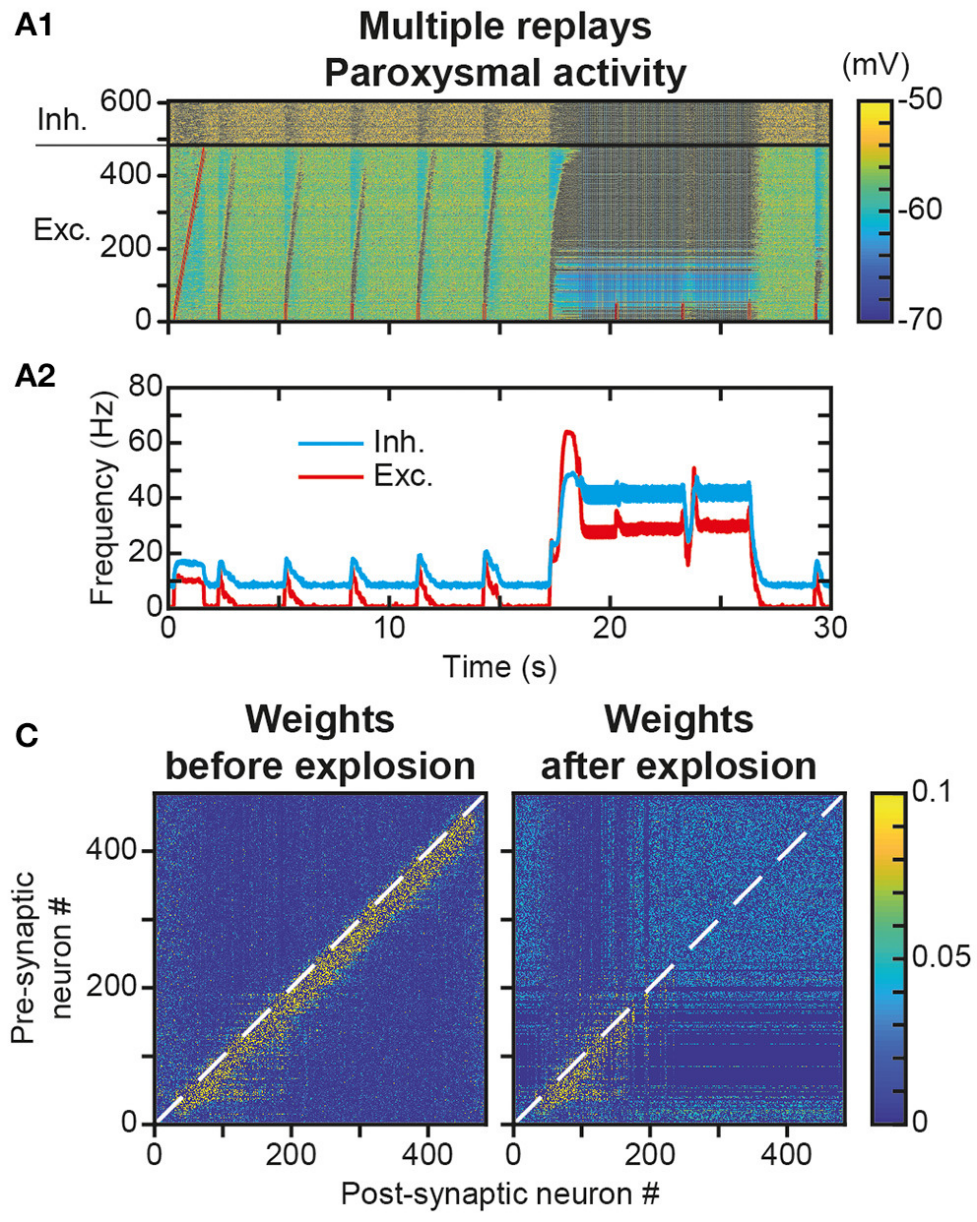
Unstable engram and network dynamics after repeated trajectory replays. **(A)**. Membrane potential of neurons in the neural network **(A1)** for 30 s during which a replay stimulus is performed on the first 50 neurons every 3 s. Mean activity of excitatory (red) and inhibitory (blue) neurons **(A2)**. **(B)**. Matrix of synaptic weights between excitatory neurons before (left) and after (right) paroxysmal network activity.

We have observed that a single replay of the trajectory only marginally modified the engram (Figure 4C vs. Figure 4A). However, we assessed whether replay repetitions could strengthen the engram significantly further. Such strengthening through repetition could compensate for the engram erasure due to spontaneous activity after the learning (Figure 6E1) and its functional consequence, the relatively rapid loss of replay capacity (Figures 6E2,E3). Intuitively, the partial increase in weight at the border of the trajectory engram after one replay (Figure 4D4 Δw_Total_, red fringes) could, after repeated replays, be strong enough to counteract the decrease observed outside replays during memorization (Figure 6D3, light blue fringe). To test this possibility, we repeated the replay stimulus every 3 s for 30 s after the presentation of the initial trajectory stimulus (Figure 7A). We observed, from the very first seconds, and even before we could test the effect of the protocol at larger timescales, that these successive stimuli, initially triggering correct trajectory replays, rapidly led to hyperactivity involving most of the neurons in the network (Figure 7A1). Such paroxysmal activity typically appeared via avalanche dynamics activating neurons at the end of the trajectory (a fraction of the network, therefore), which propagated to the whole network at increasingly higher discharge frequencies (up to tens of Hz). Moreover, this activity had an oscillatory component, visible on the time course of the frequency of the excitatory and inhibitory neurons (Figure 7A2). This paroxysmal activity partially erased the engram of the learned trajectory via synaptic scaling (Figure 7B), making it impossible to replay the trajectory following this seizure (see last stimulus, Figure 7A1), consistent with similar effects found in empirical observation during epileptic seizures (Hu et al., 2005; Meador, 2007; Truccolo et al., 2011).

### Slow Learning Stabilizes Trajectory Engram and Replays

**Figure 8:**
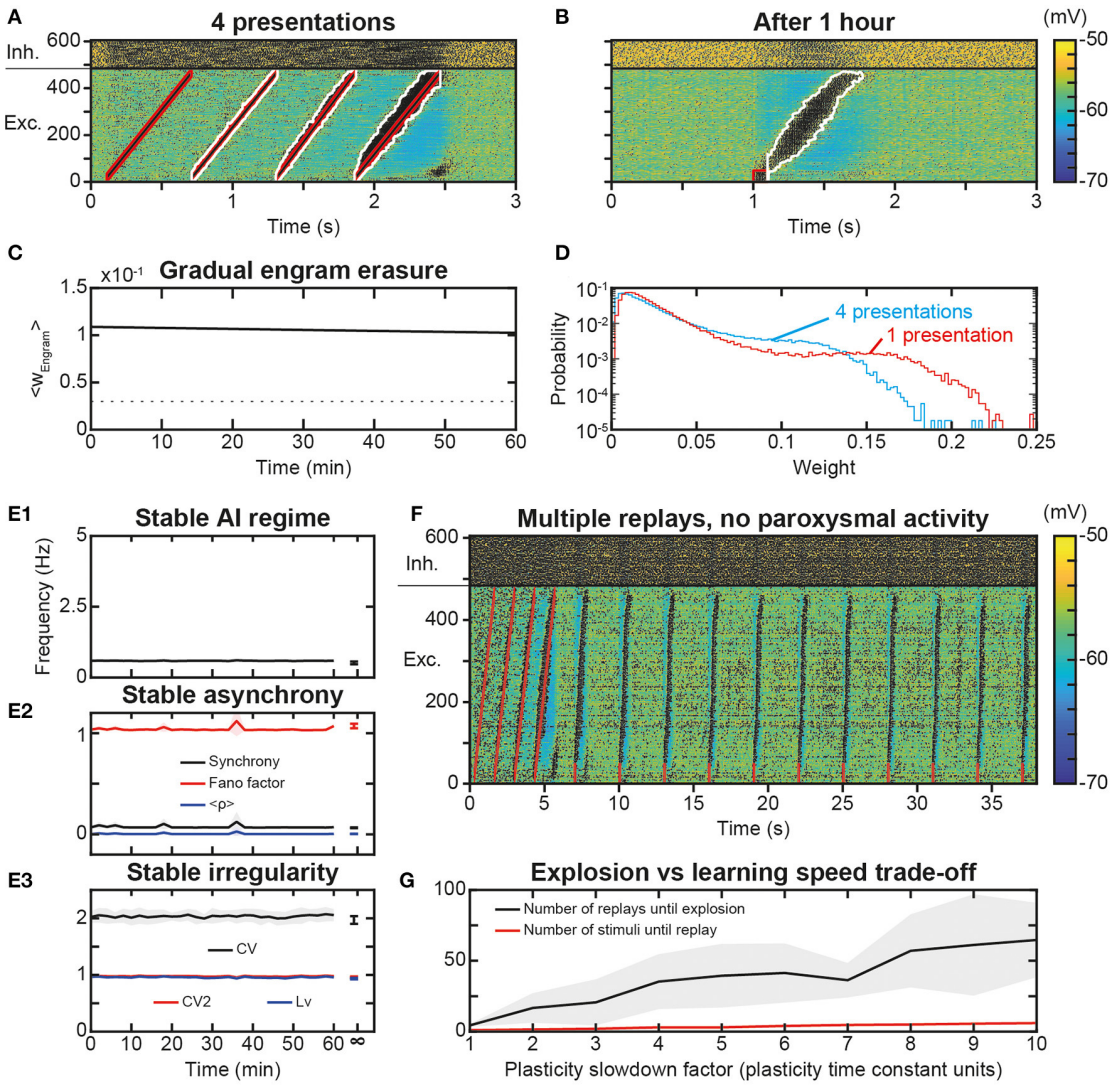
Slower learning stabilization of the engram and network dynamics. **(A)**. Membrane potential of the neural network in response to the presentation of 4 trajectory stimuli in the presence of slower STDP learning kinetics. **(B)**. Membrane voltage of the neural network for 3 s following a replay stimulation on the first 50 neurons at 1 s, 1 h after trajectory learning. **(C)**. Average weight of all engram synapses after learning for 1 h. **(D)**. Probability distribution of the synaptic weights of the excitatory synapses after 4 presentations of the trajectory stimulus during slow learning (blue), and after one presentation of the trajectory stimulus during learning with faster (standard) parameters (red). **(E)** Networks dynamics after learning with slow plasticity: frequency **(E1)**, synchrony **(E2)**, and irregularity **(E3)** of excitatory neurons. Shaded areas represent 95% confidence intervals of the mean for five network simulations. **(F)** Membrane potential of the neural network for 38 s during which a replay stimulus is performed on 50 neurons every 3 s for 10 total repetitions (as in Figure 7A) after 4 trajectory stimuli in the presence of slower STDP learning kinetics (as in Figure 8A). **(G)** Minimal number of stimulus presentations required to learn a replay (red) and maximal number of replays before paroxysmal activity (black), as a function of the plasticity slowdown factor expressed in units of plasticity standard time constant (i.e., by which slowdown factor plasticity rates are divided). The number of replays until explosion is evaluated with the same weight matrix (learned at standard plasticity speed or x1 slowdown) across different plasticity speeds, for better comparison of the effect of plasticity speeds on replay. Shaded areas represent 95% confidence intervals of the mean for 10 network simulations.

As the repetition of replay learning led to over-activation of the trajectory with plasticity speed parameters sufficiently fast for a single stimulus presentation to be learned and replayed, we investigated how slower STDP kinetic coefficients could prevent paroxysmal activity during stimulus presentations and replays. For this, we used smaller values of *K*_max_ and *P*_max_, i.e., here, divided by a factor of 6. With these values, 4 presentations of the trajectory stimulus were necessary for increasing the engram weights enough to sustain trajectory replays (Figure 8A). After such a learning protocol, the replay of the full trajectory was possible even beyond 1 h after learning (Figure 8B), whereas replay ability lasted only a few minutes with previous parameters (Figures 6E2,E3). This increase in replay memory timescale is consistent with that of the engram time constant, which was 11.5 h (Figure 8C), of the order of its theoretical estimation ~11.7 h, i.e., it was increased by a factor 6 compared to that obtained with previous parameters (1.91 and 1.95 h, respectively Figure 6E1). Remarkably, the memory of trajectory replay was increased by a factor >20 (trajectory completely replayed at >1.4 h vs. 4 min with previous parameters), so that, relatively to the timescale of the trajectory engram, the timescale for trajectory replay was further increased by a factor 3.5. Indeed, the presentation of several stimuli recruited a thicker-tailed weight distribution, with higher probability of large weights (blue curve above the red one in ~0.05–0.125; Figure 8D) but lowered probabilities of highly-weighted synapses (blue curve with negligible probabilities above 0.15; Figure 8D), because successive trajectory stimuli simultaneously evoked progressively stronger trajectory replays, recruiting more neurons at lower frequencies (Figure 8A), therefore imprinting larger engrams. Thus, slower plasticity kinetics required a larger number of successive presentations to learn the trajectory, but ensured a more robust engram involving more synapses, resulting in a better resilience to forgetting, i.e., a better quality of learning.

Finally, we assessed whether slow plasticity with multiple stimulus presentations also preserved network dynamics. AI dynamics were preserved with stable frequency (Figure 8E1), synchrony (Figure 8E2), and irregularity (Figure 8E3). We then repeated the replay stimulus every 3 s for 30 s after the presentation of the initial trajectory stimulus, a protocol which led to paroxysmal activity when considering fast plasticity. With slower kinetics, multiple replay stimuli triggered correct trajectory replays for the whole duration of the simulation (Figure 8F). We then asked whether a threshold of plasticity speed exists above which paroxysmal activity is triggered, or, conversely, the risk of paroxysmal activity linearly scales with the ability to learn fast. To do so, we parametrically explored simulations with plasticity rate divided by a slowdown factor in the range 1–10. The minimal number of stimulus presentations required to form a strong enough engram (i.e., allowing a replay) increased slowly with slower plasticity kinetics (Figure 8G, red). In parallel, the increase in the maximal number of replays before turning network dynamics into paroxysmal activity was much larger (Figure 8G, black), so that slowing plasticity kinetics increased the physiological range allowing learning while preserving network dynamics from paroxysmal activity. Hence, plasticity slow enough to preserve healthy dynamics may constitute a key constraint on the ability to learn rapidly. Furthermore, if the product of plasticity speed with the number of stimulus presentation was constant, it would indicate a linear summation of plastic effects arising from each presentation. By contrast, the number of stimulus presentations necessary for replay was lower than the factor of plasticity slowdown (5 stimuli for 10x plasticity slowdown instead of 10 stimuli, Figure 8G). This is due to successive stimulations overlapping with replays (i.e., stimulus presentations after the first one induce replays, Figure 8A), suggesting progressive facilitation of learning at slow plasticity speeds.

## Discussion

Here, we show that it is possible to learn neural trajectories (dynamical representations) using a spike timing-dependent plasticity (STDP) learning rule in local PFC circuits displaying spontaneous activity in the asynchronous irregular (AI) regime. We used a physiological model of plasticity (Delord et al., 2007; Graupner and Brunel, 2012; He et al., 2015) continuously occurring online, i.e., without decoupling simulations of learning and activity. Presentation of a dynamic stimulus, the trajectory, resulted in the writing of a synaptic engram of the trajectory on a rapid timescale (seconds), as well as its long-term storage at the timescale of the order of several hours. The network replayed the trajectory upon stimulation of a subset of the engram at the timescale of the order of dozens of minutes. These results indicate that disordered AI activity does not necessarily jeopardize the encoding and replay of neural trajectories. Conversely, the weak but continuous plastic processes that noisy AI produces did not erase the synaptic engram of neural trajectories, at least before several hours. In turn, the learning of a trajectory engram within network synapses was not found to alter the AI characteristics of PFC activity. From a functional perspective, we show that trajectory activity accounted for both types of dynamics subserving working-memory in the PFC, i.e., persistent activity (Constantinidis et al., 2018) and dynamical coding (Lundqvist et al., 2018), and help understanding how they can be reconciled (Murray et al., 2017; Cavanagh et al., 2018; Enel et al., 2020). Together, these results offer a consistent theoretical framework accounting for how dynamical representations can be learned, memorized and replayed in PFC circuits in physiological conditions.

This model was built to reproduce functional phenomenology of the PFC (learning, replays at different timescales, dynamic or persistent coding, see below), based on biophysical constraints from the experimental literature at the molecular, cellular and network levels, rather than by artificial training. If overall architectural properties of the model are observed in the PFC, such properties are also compatible with other non-prefrontal cortices with trajectory replays, lending strength to the genericity of the current study's results. For example, the excitatory/inhibitory network balance, observed in the PFC (Shu et al., 2003; Haider et al., 2006), is also observed and essential to computations across non-PFC structures (Isaacson and Scanziani, 2011). Similarly, the over-representation of bidirectional connections in the PFC (Wang et al., 2006) is a general property in cortical networks (Song et al., 2005). While the PFC has been less subject to the investigation of synaptic scaling compared to other structures, its presence across many non-PFC cortical structures (for e.g., sensory cortices, hippocampus, motor cortex) and crucial role for synaptic learning stabilization (Keck et al., 2017) makes it a plausible mechanism in PFC. Certain lines of evidence suggest its presence in PFC (Wang and Gao, 2012; Sweatt, 2016), although further confirmation is needed.

In the model, external feedforward inputs are constant, as in previous models of characteristic PFC activity (for e.g., Brunel, 2000). Therefore, the variability of neuronal discharge observed in the network entirely arises from internal dynamics among recurrent connections, as the network is in the asynchronous irregular regime (Destexhe et al., 2003; London et al., 2010). It would be interesting to study versions of the model with feedforward inputs variability, as occurring in real PFC circuits. However, this option was out of scope as we focused on the internal interactions between the spontaneous AI regime, learning processes affecting the synaptic matrix and trajectory replays. As another potential extension to our study, one could explore the influence of rhythmic inputs from the hippocampus (theta rhythms, Siapas et al., 2005; Benchenane et al., 2011) or from the olfactory pathways (delta rhythms, Moberly et al., 2018), which are known to be important for behaviorally-relevant neural activity and memory replays.

### Molecular Plasticity and Memory in the PFC

In the PFC, e-STDP necessitates more than the pre-post synaptic pairings used in spike-timing protocols, as long-term potentiation (t-LTP) emerges in the presence of dopaminergic or cholinergic tonic neuromodulation, or when inhibitory synaptic transmission is decreased (Couey et al., 2007; Xu and Yao, 2010; Ruan et al., 2014). Moreover, Hebbian STDP (i.e., t-LTP for pre-then-post and t-LTD for post-then-pre spiking) is observed when followed by phasic noradrenergic, dopaminergic or serotoninergic neuromodulation (He et al., 2015). Hence, we assumed that t-LTP and t-LTD co-exist, and STDP is thus Hebbian, in the PFC of behaving animals, where both phasic and tonic neuromodulation are encountered during behaviorally relevant learning (Dembrow and Johnston, 2014). The present study did not incorporate noradrenergic, serotoninergic and dopaminergic transformation of eligibility traces into effective plastic modifications found at PFC excitatory synapses (He et al., 2015), a possible substrate of context- and reward-modulated learning in PFC circuits (Ellwood et al., 2017). The present work also did not consider alternative biophysical processes that may participate to sculpt dynamical and flexible neural representations in the PFC (Buonomano and Maass, 2009; Stokes, 2015). For instance, short-term synaptic plasticity (Mongillo et al., 2008) may affect network dynamics through slow hidden (e.g., biochemical) variables. Such a silent-based coding of past activity could possibly account for the near-complete disappearance of activity observed sometimes during working memory (Stokes, 2015) and its interaction with activity-based working-memory in the PFC (Barbosa et al., 2020) remains to be elucidated. Similarly, inward current-mediated bistability such as with persistent sodium, or calcium-activated non-specific currents (Delord et al., 1997; Rodriguez et al., 2018), can produce cellular forms of memory that may take part in dynamic representations in the PFC, either through retrospective memory of past information or in prospective computations of forthcoming decisions and actions. Finally, the present study did not consider anti-homeostatic forms of intrinsic plasticity (i.e., the plasticity of intrinsic properties) which may represent an essential mean to learn and regulate dynamic representations (Zhang and Linden, 2003).

### Stable Spontaneous AI Dynamics in the PFC in the Presence of Plasticity and Learning

Hebbian forms of plasticity (Abbott and Nelson, 2000), such as the STDP of excitatory synapses (Markram et al., 2012) modeled here, increase weights between neurons that are frequently co-activated. Stronger synapses potentiated by STDP, in turn, statistically increase the frequency of future co-activations. These rules thus constitute positive feedback loops (anti-homeostatic) between activity and connectivity. As a consequence, synaptic runaway (Keck et al., 2017; Zenke et al., 2017) produces network instability toward saturated or quiescent activity and connectivity. In recurrent network models, synaptic plasticity typically decreases the dynamics complexity toward regular activity such as limit-cycle or quasi-periodic attractors (Morrison et al., 2007; Siri et al., 2007; Litwin-Kumar and Doiron, 2014) that resembles neural dynamics encountered during sleep or paroxysmal crises. However, activity in the PFC and other cortices during wakefulness is characterized by asynchronous irregular spiking at low frequency (Ecker et al., 2010; Renart et al., 2010), due to the balance between strong excitatory and inhibitory synaptic currents (Destexhe et al., 2003). AI spiking is compatible with critical or even chaotic dynamics (Beggs and Plenz, 2003; Hahn et al., 2010; London et al., 2010), which may benefit temporally complex computations (Bertschinger and Natschläger, 2004) believed to be performed by the PFC (Compte, 2003).

Many studies show that e-STDP rules are deleterious to AI dynamics such that compensating homeostatic mechanisms are required to control neuronal activity, for e.g., a metaplastic e-STDP rule with sliding-threshold (Boustani et al., 2012), synaptic scaling (which keeps the sum of pre-synaptic excitatory weights constant, Zenke et al., 2013), STDP of inhibitory synapses (i-STDP; ensuring excitation-inhibition balance, Vogels et al., 2011) or intrinsic plasticity of ionic conductances (regulating action potential threshold, Naudé et al., 2013). In the present detailed biophysical model, we found that a combination of e-STDP where all pre-/post- pairings were taken into account (all-to-all STDP), together with synaptic scaling, preserves AI dynamics. All-to-all e-STDP without scaling can also preserve AI dynamics, but at the price of unstable fluctuating synaptic weights (Morrison et al., 2007), while weight distributions were stable here. Moreover, the present study shows that network stability held not only with random recurrent connections, but also in the presence of an engram involving a significant fraction of strong, potentiated synapses in all excitatory neurons. In the absence of synaptic scaling, learning static patterns into synaptic engrams with e-STDP disrupts AI dynamics toward pathological high-frequency oscillations (Morrison et al., 2007; Litwin-Kumar and Doiron, 2014), or with i-STDP leads to AI activity with unrealistic high firing frequency states and sharp state transitions (Litwin-Kumar and Doiron, 2014), at odds with PFC dynamics in awake animals (Compte, 2003). A metaplastic form of e-STDP conserves AI dynamics on a short-timescale (one second) but AI stability remains unchecked at longer timescales (Boustani et al., 2012). This is only the case with static stimulus, as learning receptive fields using dynamical stimulus leads to a catastrophic decrease in the complexity of the AI regime (Boustani et al., 2012). Altogether, our study thus suggests that synaptic scaling represents a more efficient form of homeostatic compensation (rather than metaplastic e-STDP, or i-STDP) for learning trajectory engrams without the deleterious effects of STDP disrupting AI dynamics. We used here an instantaneous synaptic scaling, because our model, like most models, requires synaptic scaling at faster or equal timescales than synaptic plasticity for stable learning, far from the experimentally observed homeostatic or metaplastic timescales of hours to weeks (Zenke et al., 2017). This constraint suggests the existence of as yet unidentified rapid compensatory processes, potential candidates being heterosynaptic plasticity (Fiete et al., 2010), intrinsic plasticity (Zhang and Linden, 2003; Naudé et al., 2013), input normalization by feed-forward inhibition (Pouille et al., 2009; Keck et al., 2012), and the implication of astrocytes (Papouin et al., 2017). Additionally, at slower timescales, sleep-dependent consolidation mechanisms may provide global compensatory synaptic down-scaling offline (Tononi and Cirelli, 2003).

### Learning Dynamical Representations in the PFC Under AI Dynamics

Phenomenological e-STDP models fail to learn engrams in noisy AI states because of their sensitivity to spontaneous activity. The absence of STDP weight-dependence forbids learning and induces the direct loss of engrams (Boustani et al., 2012), while without synaptic scaling, learning fails with catastrophic consequences in terms of network dynamics (see above; Morrison et al., 2007). A weight-dependent e-STDP rule endowed with homeostatic metaplasticity (instead of synaptic scaling, as here) allowed learning the engram of a presented stimulus while preserving AI dynamics, although it unrealistically left neurons of the engram in a state of permanent activity (Boustani et al., 2012). Likewise, i-STDP enables learning of engrams, but with unrealistic AI activity (see above; Litwin-Kumar and Doiron, 2014). Here, we find that the combination of a weight-dependent Hebbian e-STDP rule and synaptic scaling allows for the learning of engrams in local PFC recurrent networks under conditions of AI dynamics, as found in behaving mammals.

Phenomenological STDP models based on neighboring spike-doublet or spike-triplet schemes often produce side effects (either sensitivity to noisy activity, or runaway plasticity) due to the temporal bounds of the pre- and post-couplings they consider (Boustani et al., 2012). The present STDP model describes continuous post-synaptic biophysical dynamics that account for all pre-/post-pairings (all-to-all STDP) and is thus more realistic than phenomenological STDP models. Here, the temporal asymmetry of the spike-timing dependence of the e-STDP rule arises from a detailed description of calcium dynamics. Calcium arises from two different sources of calcium that originate from the influence of AMPA, NMDA and VDCC channel activations (see *Materials and Methods*; Graupner and Brunel, 2012), which accounts for the relative influence of pre-synaptic evoked excitatory post-synaptic potentials and of backpropagating post-synaptic activity. However, this rule remains simple compared to models describing more complete signaling scenarios (Manninen et al., 2010), allowing simulation at the network scale.

In feed-forward networks endowed with this STDP rule, and for conditions of spiking frequency and irregularity similar to AI activity, plastic modifications essentially depend on firing frequency rather than on the precise timing of spikes, because equivalent probabilities of encountering pre-then-post and post-then-pre spike pairs in conditions of stationary spiking essentially blurs net spike-timing effects (Graupner et al., 2016). Moreover, t-LTP dominates t-LTD, because t-LTD is multiplicative (Bi and Poo, 1998; van Rossum et al., 2000), i.e., scaled by weak weight values (Graupner et al., 2016). Consistent with these observations, in the present PFC recurrent network model, plasticity was essentially frequency-dependent under conditions of stationary spiking, and t-LTP dominated t-LTD under spontaneous AI dynamics, being principally compensated by synaptic scaling. However, during trajectory presentation or trajectory replay, i.e., when pre-post spiking was enforced to be temporally asymmetric, t-LTD nevertheless contributed to compensate t-LTP and determined overall resulting modifications on the same order than scaling.

The previous studies that have addressed the possibility of engram learning in recurrent networks with AI dynamics focused on static stimuli (Morrison et al., 2007; Boustani et al., 2012; Litwin-Kumar and Doiron, 2014). By contrast, our study demonstrates engram learning and activity replay of dynamical stimuli, such as the sequences or trajectories of activity that occur during cortical AI dynamics in behaving animals (Kaefer et al., 2020). Standard static Hebbian assemblies, which learn static stimuli through strong bidirectional connections between neurons of the assembly and replay the static activity through pattern completion, induce avalanche-like convergent dynamics toward a static attractor, which are too low-dimensional to account for physiological data. Remarkably, the present study demonstrates the possibility for engrams of dynamic stimuli in the disordered AI state, despite the fact that they relied on mono-directional strengthening of synaptic connections, which favors propagation of activity, but does not allow for the convergent effect of static patterns and the positive feedback inherent to it.

### Long-Term Memory of Dynamical Representations in the PFC Under AI Dynamics

The present study underlines the importance of slow plasticity kinetics together with repeated presentations for learning dynamic representations in PFC networks. Faster kinetics allowed one-shot learning of trajectory engrams, but extensive training could then induce paroxysmal activity during the trajectory replays that partly erased the engram, which was ultimately detrimental to the learning and replay process. This synchronous increase in neuronal activity in the model is reminiscent of epileptic seizures (Truccolo et al., 2011), which have been found to cancel out the plasticity effects of synaptic weights (Hu et al., 2005), and affect memory (Meador, 2007), as we found here. By contrast, slower kinetics resulted in more stable engrams, while highlighting the importance of repeated presentations of the dynamic stimulus, similarly to observations with static patterns (Boustani et al., 2012). Parametric exploration of plasticity kinetics showed a tradeoff between the number of stimulus repetitions required to form an engram and the risk of paroxysmal activity. However, slowing down plasticity decreased the risk of over-activation while preserving the ability to learn fast (even though not through one-shot learning). Consistent with our results, learning occurs gradually in the PFC, and at a slower pace than in the hippocampus and basal ganglia (Pasupathy and Miller, 2005; Buschman and Miller, 2014). The tradeoff between fast learning and paroxysmal risk may constitute a constraint for the PFC, with the preservation of asynchronous irregular dynamics preventing one-shot learning based on synaptic plasticity alone. One-shot learning, which occurs in well-trained animals, may thus require additional mechanisms for structural learning (Gallistel and Matzel, 2013).

Fast learning together with stable memory is considered in many synaptic plasticity models to rely on auto-phosphorylation of the calmodulin-dependent protein kinase II (CaMKII). CaMKII auto-phosphorylation is appealing because it constitutes a positive-feedback loop (inducing fast plasticity) underlying bistable dynamics (providing infinite memory of a single potentiated synaptic state). However, we did not consider CaMKII in the present model, because CamKII is not necessary to the maintenance of synaptic modifications (Chen et al., 2001; Lengyel et al., 2004). Moreover, activity-dependent synaptic modifications are not systematically bistable (i.e., they can be graded; Montgomery and Madison, 2002; Tanaka et al., 2008; Enoki et al., 2009) and they can fade with time scales from seconds to minutes (Hempel et al., 2000).

Here, the stability of molecular memory originated from extremely slow synaptic weight dynamics, resulting in slow exponential forgetting of the engram. Slow weight dynamics arose from activity-dependent kinase and phosphatase (aKP, Delord et al., 2007), which are weakly activated at near-basal calcium concentrations associated with low spiking frequency during AI dynamics. Such aKP signaling processes are ubiquitous (e.g., PKA, PKC, calcineurin) and confer an activity-dependent control over the rate of plasticity and memory (Delord et al., 2007), which is essential for flexible learning in the PFC (Fusi et al., 2005). Alternatively, when implemented with low copy molecule numbers at individual synapses, bistable models faced with noise also exhibit exponential forgetting of memory when averaged over synapses and trials (Fusi et al., 2005). Here, the memory of the trajectory engram admitted an effective time constant of the order of 2 h in network simulations, consistent with its theoretical prediction (see *Materials and Methods*), but longer memories could be expected for lower values of *P*_max_ and *K*_max_, the maximum phosphatase and kinase activations. However, the time constant for plasticity would also increase, slowing learning too, while its current value is compatible with induction times of synaptic plasticity (Malenka et al., 1992). Alternatively, a higher calcium phosphatase half-activation (*P*_*Ca*_), which is physiologically possible (Delord et al., 2007), would allow for a longer memory timescale while preserving rapid learning (at large calcium, the time constant of plasticity is independent of *P*_*Ca*_). Hence, specifying biophysical models with precise kinetic parameters is essential because they have huge consequences on the stability of network dynamics, learning and the time scale of memory (Zenke et al., 2013). Specifically, homeostatic scaling appeared important here as for learning, since its absence was reported to forbid the memory of static patterns in recurrent network models because of catastrophic forgetting due to fluctuating synaptic weights (Morrison et al., 2007).

The timescale of trajectory replay scaled with that of the engram. This is because replay requires a sufficiently preserved engram to emerge from synaptic interactions between neurons. However, the lifetime of trajectory replay was an order of magnitude smaller than that of the trajectory engram, because replay requires neuronal interactions that are non-linear and therefore sensitive to decreases in synaptic weights. Interestingly, the long-term degradation of trajectory replay was due to incomplete replay at the end of the trajectories learned, in a manner consistent with the primacy effect of medium-term learned sequences (Greene et al., 2000). Besides, the memory of trajectory replay did not only rely on biophysical parameters but also on the learning protocol. Indeed, slower learning with repetitions increased the quality of engram by better anchoring the learned trajectory, through a larger number of synapses. Slow plasticity of a large number of synapses from a recurrent network, through repetition, may thus underlie the robustness of PFC-dependent memories (Buschman and Miller, 2014). In addition to extensive training, the maintenance of trajectory engrams over longer timescales may be reached by regular replays, as observed in PFC-dependent active executive processes such as trajectory reactivations (Stokes, 2015), spontaneous replays (Kaefer et al., 2020), rehearsal and refreshing (Raye et al., 2007), or consolidations (Dudai, 2012). At the molecular scale, the possibility of synaptic tagging could be incorporated in the model (Clopath et al., 2008) in order to stabilize the engram and account for longer memory timescales.

Humans or animals generally learn complex navigational paths such as sensory, motor or behavioral sequences in a progressive manner. Thus, PFC circuits are often challenged with the necessity to process several parts of whole neural trajectories that are discovered as sequences of elementary parts encountered at separate points in time. Moreover, prospective processes in the PFC require recombining elementary neural trajectories into new trajectory representations serving the planning of future actions, choices or navigational paths, for e.g., during rule switching and behavioral adaptation (Ito et al., 2015; Mashhoori et al., 2018; Kaefer et al., 2020). Besides, sequences of non-spatial items have been shown to be processed in a spatial frame in primates (Jensen et al., 2013), likely involving neural trajectories. We found that STDP-based trajectory learning and replay in the network was able to learn trajectory fragments, transitions between fragments, and to chunk them into a whole trajectory, as found in the PFC (Ostlund et al., 2009; Dehaene et al., 2015). Moreover, the network displayed the ability to reconstitute a whole trajectory (i.e., a macroscopic sequence) based on trajectory fragments (i.e., overlapping microscopic sequences), independently of their order of presentation, i.e., to acquire ordinal knowledge about sequences of trajectory fragments (Jensen et al., 2013; Dehaene et al., 2015). However, STDP-based trajectory learning in our PFC network model was unable to learn higher-order representations of algebraic patterns or more complex nested structures (Dehaene et al., 2015), or to categorize sequences into specific classes (Shima et al., 2007). Assessing such possibilities using more elaborated, reward-dependent, forms of STDP learning rules might deserve future explorations.

### Multiple Functional Relevance of STDP-Based Neural Trajectories in the PFC

We found in our model that the same network, taught with the same stimulus, could generate a large range of replay duration and compression factors, including those characterizing regular (Batuev, 1994; Fujisawa et al., 2008; Cromer et al., 2010; Mante et al., 2013; Yang et al., 2014; Ito et al., 2015; Markowitz et al., 2015; Schmitt et al., 2017; Tiganj et al., 2017; Nakajima et al., 2019; Passecker et al., 2019; Enel et al., 2020) and fast (Jadhav et al., 2016; Tiganj et al., 2017; Mashhoori et al., 2018; Yu et al., 2018; Shin et al., 2019; Kaefer et al., 2020) timescale replays in behaving animal. We found that the time constant of NMDA decay dynamics was essential in controlling the duration and compression factor of trajectory replays. In PFC circuits, dopamine slows decaying dynamics of NMDA-mediated EPSPs through D1-receptors (Chen et al., 2004; Onn et al., 2006) in an almost instantaneous manner (Onn and Wang, 2005). In addition to dopaminergic regulation, other forms of neuromodulation affect NMDA dynamics (Lutzu and Castillo, 2021). Our results suggest that rapid and bidirectional regulation of biophysical parameters in PFC networks by ongoing neuromodulation—as attentional demands and reward outcomes vary at the trial timescale—may control replay duration, compression factors, and the relative rate of regular vs. fast timescale replays.

Besides, individual neuronal activity displayed lower firing frequency during replay compared to the activity induced by the stimulus, consistent with sparse coding of representations after learning. Firing rates of individual neurons during stimuli or delays in working memory tasks, as well as in navigation tasks, vary considerably across species and behavioral contexts, spanning two orders of magnitude from ~1 to ~ 100 Hz (Fuster and Alexander, 1971; Batuev, 1994; Romo et al., 1999; Baeg et al., 2003; Yang et al., 2014; Markowitz et al., 2015; Tiganj et al., 2017). Frequencies of dozens Hz are common in individual PFC neurons (Funahashi et al., 1989; Romo et al., 1999; Brody et al., 2003; Fujii and Graybiel, 2003; Shinomoto et al., 2003; Jun et al., 2010; Tiganj et al., 2017; Enel et al., 2020). In the present model, frequencies of individual neurons were actually ~100 Hz during stimuli and presentations, and 20–60 Hz during replays (Figures 5B1,B3). Thus, although larger than those observed during stimuli, individual frequencies were globally of the order of magnitude of those empirically observed. Mean frequencies in our network ranged below 10 Hz (Figures 5B1,B3), (7A2), in accord with experimental literature (Funahashi et al., 1989; Romo et al., 1999; Brody et al., 2003; Fujii and Graybiel, 2003; Shinomoto et al., 2003; Jun et al., 2010; Tiganj et al., 2017; Enel et al., 2020).

In the PFC, representations for executive functions and cognition can present less explicit dynamic coding schemes than regular timescale neural trajectories presented here. For instance, working memory can display intricate patterns of complex (heterogeneous but non-random) dynamic activities that can hardly be disentangled into simpler well-separate transient patterns of activity (Jun et al., 2010). However, during working memory tasks, PFC persistent delay activity is selective and maintains online content-specific representations. Working memory does often, but not systematically, require underlying persistent activities, often in a stable activity state (Goldman-Rakic, 1995; Compte et al., 2000; Durstewitz et al., 2000; Wang, 2001; Constantinidis et al., 2018). It can also rely on dynamical sequences of activities disappearing and reappearing, depending on instantaneous computational task-relevant requirements (Sreenivasan et al., 2014; Stokes, 2015; Lundqvist et al., 2018). The coexistence of stable population coding together with heterogeneous neural dynamics has been observed in the PFC during working memory tasks (Murray et al., 2017).

Here, trajectory replays offer a possible unified framework that can participate to reconcile opposite views regarding the nature of information persistent vs. dynamic coding in the PFC (Constantinidis et al., 2018; Lundqvist et al., 2018). Indeed, we find that while individual neurons displayed transient (hundreds of milliseconds) overlapping bumps of activity, implementing a “relay race” form of explicit dynamic coding (Batuev, 1994; Brody et al., 2003; Cromer et al., 2010; Yang et al., 2014; Schmitt et al., 2017), their population activity persisted at the second timescale, ensuring the maintenance of the representation across time (Murray et al., 2017; Cavanagh et al., 2018; Enel et al., 2020). Depending on the functional context, neural trajectories learned here could be interpreted as the actual explicit representation of a trajectory unfolding online, granted that the decoding downstream neural structure can resolve individual activities of the network. Alternatively, if the downstream decoding neural structure only globally decodes the population average of network dynamics, activity would then be interpreted as an integrated and stable persistent representation of the trajectory as a whole (i.e., as a symbolic entity). This dichotomy is congruent with that found in the PFC, whereby individual neurons encode information at short timescales while the population as a whole persistently maintains information at longer time scales (Meyers et al., 2008). In this scheme, working memory representations would rely on individual neurons collectively stabilizing a dynamic population-level process (Murray et al., 2017; Cavanagh et al., 2018; Enel et al., 2020).

Interestingly, we found that the population activity of trajectory replays accounted for the decreasing pattern of activity that can be observed in the PFC (Cavanagh et al., 2018; Enel et al., 2020). Trajectory replays also displayed strong variability, as observed in the PFC during delay activities (Compte, 2003; Shafi et al., 2007). While within-trial variability across neurons essentially came from the fact that neurons spiked at distinct periods along the trajectory, inter-trial variability for each neuron originated from the noisy AI dynamics. Inter-trial variability accumulated over time for neurons situated later in the trajectory, henceforth the temporal tuning of neurons widened with their position in the sequence (Tiganj et al., 2017).

## Data Availability Statement

The raw data supporting the conclusions of this article will be made available by the authors, without undue reservation.

## Author Contributions

MS, JV, JN, and BD developed the model. MS, JV, DM, and BD contributed to numerical simulations and their analysis. MS, JV, DM, JN, and BD wrote the article. All authors contributed to the article and approved the submitted version.

## Conflict of Interest

The authors declare that the research was conducted in the absence of any commercial or financial relationships that could be construed as a potential conflict of interest.
